# Genomic prediction of starch content and chipping quality in tetraploid potato using genotyping-by-sequencing

**DOI:** 10.1007/s00122-017-2944-y

**Published:** 2017-07-13

**Authors:** Elsa Sverrisdóttir, Stephen Byrne, Ea Høegh Riis Sundmark, Heidi Øllegaard Johnsen, Hanne Grethe Kirk, Torben Asp, Luc Janss, Kåre L. Nielsen

**Affiliations:** 1 Department of Chemistry and Bioscience, Aalborg University, Fredrik Bajers Vej 7H, 9220 Aalborg, Denmark; 2 Department of Molecular Biology and Genetics, Aarhus University, Forsøgsvej 1, 4200 Slagelse, Denmark; 3 LKF Vandel, Grindstedvej 55, 7184 Vandel, Denmark; 4 Department of Molecular Biology and Genetics, Aarhus University, Blichers Allé 20, 8830 Tjele, Denmark; 5 Present Address: Crop Science Department, Teagasc, Oak Park, Carlow, Ireland

## Abstract

*****Key message***:**

**Genomic prediction models for starch content and chipping quality show promising results, suggesting that genomic selection is a feasible breeding strategy in tetraploid potato.**

**Abstract:**

Genomic selection uses genome-wide molecular markers to predict performance of individuals and allows selections in the absence of direct phenotyping. It is regarded as a useful tool to accelerate genetic gain in breeding programs, and is becoming increasingly viable for crops as genotyping costs continue to fall. In this study, we have generated genomic prediction models for starch content and chipping quality in tetraploid potato to facilitate varietal development. Chipping quality was evaluated as the colour of a potato chip after frying following cold induced sweetening. We used genotyping-by-sequencing to genotype 762 offspring, derived from a population generated from biparental crosses of 18 tetraploid parents. Additionally, 74 breeding clones were genotyped, representing a test panel for model validation. We generated genomic prediction models from 171,859 single-nucleotide polymorphisms to calculate genomic estimated breeding values. Cross-validated prediction correlations of 0.56 and 0.73 were obtained within the training population for starch content and chipping quality, respectively, while correlations were lower when predicting performance in the test panel, at 0.30–0.31 and 0.42–0.43, respectively. Predictions in the test panel were slightly improved when including representatives from the test panel in the training population but worsened when preceded by marker selection. Our results suggest that genomic prediction is feasible, however, the extremely high allelic diversity of tetraploid potato necessitates large training populations to efficiently capture the genetic diversity of elite potato germplasm and enable accurate prediction across the entire spectrum of elite potatoes. Nonetheless, our results demonstrate that GS is a promising breeding strategy for tetraploid potato.

**Electronic supplementary material:**

The online version of this article (doi:10.1007/s00122-017-2944-y) contains supplementary material, which is available to authorized users.

## Introduction

Potato (*Solanum tuberosum* L.) is the third most important food crop worldwide after wheat and rice with 385 million tonnes fresh weight of tubers produced in 2014 from 19.2 million hectares of land (FAOSTAT 2015). In addition, it is the most efficient producer of food energy and nutrition per unit area with similar or less input of nutrients and water compared to cereals (FAOSTAT 2015). In a future agricultural scenario, where more food has to be produced from less area, these characteristics make potato an interesting crop.

To meet the demands of farmers, industry, and consumers, potato breeding seeks to develop improved varieties, which combine higher yield with tuber traits optimised for the various end uses and not least resistance to pests and diseases. Important quality traits for potato include starch content and low accumulation of reducing sugars during storage for the production of potato chips (crisps) and French fries. Following water, starch is the major constituent of the tuber, and is used in a variety of food and non-food products. In the EU, 18% of potato production is used for starch production, but in some countries, this may be higher, e.g., in Denmark up to 60% is used for starch extraction (Birch et al. 2012). For these applications, high starch content is desirable. In contrast, for the fresh potato market, lower to medium starch content is desirable. In the potato chips and French fries processing industry, the most important quality trait of potato tubers is the content of reducing sugars in the tuber upon cold storage. At the high temperatures during frying, reducing sugars undergo the non-enzymatic Maillard reaction with amino acids, resulting in dark coloured and bitter products and the production of carcinogenic acrylamides (Shallenberger et al. 1959; Medeiros Vinci et al. 2012). The amount of reducing sugars increases during the cold storage necessary to inhibit sprouting and reduce waste (Isherwood 1973).

Today, potato breeding is largely conducted through classical selective breeding involving crosses between pairs of parents followed by years of evaluation and selection. The selection cycle from an initial cross to a novel variety release requires 10–15 years (Halterman et al. 2016). Furthermore, multiple difficulties are associated with potato breeding, resulting in slow breeding gain. Indeed, the yield of cereals per area worldwide has increased by 190% in the period between 1961 and 2014, while the yield of potatoes in the same period has only increased by 60% (FAOSTAT 2015). This is mostly due to the fact that most potato cultivars are autotetraploid, so deleterious alleles are ineffectively eliminated from the gene pool over a number of generations that is relevant in breeding. In addition, the elite population is extremely diverse (Tomato Genome Consortium et al. 2012) and highly heterozygous, making prediction of performance of new cultivars particularly difficult (Potato Genome Sequencing Consortium 2011).

Progeny testing has demonstrated genetic gains in potato breeding (Bradshaw et al. 2009), and more recently, the use of phenotypic best linear unbiased prediction (BLUP) estimated breeding values has demonstrated a clear advantage over both phenotypic recurrent selection and progeny testing (Slater et al. 2014b). In addition, the use of molecular markers provides the opportunity to improve breeding significantly, and marker-assisted selection (MAS) has the ability to select for traits several years earlier in a program than would be practical using conventional screening methods. Several attempts have been made to implement MAS in potato breeding, but its overall impact on improving potato breeding efficiency has been limited. Due to the complexities of highly diverse autotetraploid genetics and the difficulties connected to linkage studies in tetraploid potato (Slater et al. 2014a, b), MAS is primarily used in potato to select for single dominant traits such as late blight resistance (Rizza et al. 2006; Ottoman et al. 2009; Ortega and Lopez-Vizcon 2012; Schultz et al. 2012). When it comes to quantitative traits such as yield, starch content, and frying colour, progress has been minimal, although a number of allelic variants influencing these traits have been identified recently (Li et al. 2013; Schönhals et al. 2016). MAS is considered best suited for traits with a few major-effect genes, and not for traits where the genetic variation is the results of a large number of loci of small effect (e.g., yield) (Heffner et al. 2009). In contrast, genomics-assisted breeding methods such as genomic selection (GS) does not have this limitation (Meuwissen et al. 2001; Heffner et al. 2009; Jannink et al. 2010). GS is a form of MAS that predicts breeding values of individuals based on genome-wide molecular markers. It is assumed that all quantitative trait loci (QTL) are in linkage disequilibrium with at least one marker and that all the genetic variance can be explained by the markers (Goddard and Hayes 2007). GS is thus considered to be particularly promising for predicting quantitative, complex traits controlled by multiple small effect loci, such as starch content and chipping quality (van Eck 2007; Li et al. 2008, 2013; Fischer et al. 2013; Schreiber et al. 2014). Marker effects are estimated from phenotypes and genotypes of a training population and are then used to calculate genomic-estimated breeding values (GEBVs) of a breeding population, using only genotypic data. Good breeding candidates can be selected based on GEBVs before being tested in field experiments, potentially reducing the breeding cycle (Heffner et al. 2009; Heffner et al. 2010; Jannink et al. 2010; Slater et al. 2016).

In this paper, we describe the results of genomic prediction of tetraploid potato for two important quality traits; starch content and chipping (crisping) quality. We applied genotyping-by-sequencing (GBS) to a population of 762 individuals, called the MASPOT population, and estimated GEBVs using linear regression models. We compared prediction accuracies of three statistical models: genomic best linear unbiased prediction (GBLUP), BayesA, and BayesC. Cross-validation was used to determine the robustness of GEBVs. In addition, a test panel of 74 breeding clones unrelated to the MASPOT population was selected and genotyped for model validation. Furthermore, to benchmark the performance of GS, a genome-wide association study (GWAS) was performed to select significant markers associated with the traits in question which were then used for prediction.

## Materials and methods

### Plant material

**Fig. 1:**
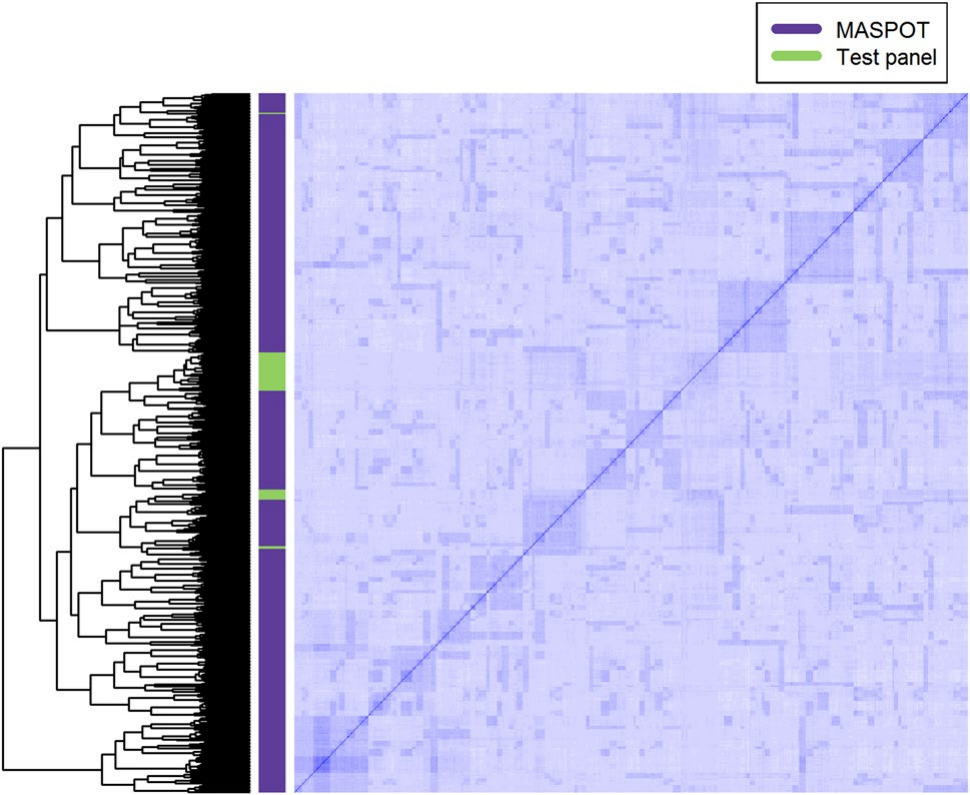
Heat map of the genomic relationship matrix for the 755 offspring in the MASPOT population (*purple* marking in the *left* panel) and the 63 individuals in the test panel (*green* marking). The matrix is obtained from 171,859 markers. *Rows* and *columns* represent each individual. The absence of obvious high intensity off-diagonal clusters indicates absence of population structure

762 clones were randomly chosen from a mapping population established at the breeding station in Vandel, Denmark, called the MASPOT population. The MASPOT population consists of roughly 5000 offspring that were generated by systematic cross pollination of 18 distinct potato cultivars in a full diallel crossing design, either established varieties or advanced breeding clones (see Online Resource 1 for a detailed description). The selected subset of 762 offspring is referred to as the MASPOT population in this paper. The offspring were planted in field trials at Vandel, Denmark in 2013 and 2014 in duplicates. Plant density was approximately 40,000 plants/hectare with 30 cm between plants and 75 cm between rows. In 2013, the offspring were planted 24–25 April and harvested 12–30 August. The tubers were desiccated 1–2 weeks before harvesting. In 2014, the offspring were divided in four groups based on earliness of parents. The groups were planted 24, 25, 28, or 29 April and harvested 11–29 August, also with 1–2 weeks of desiccation, where group 1 was harvested first and group 4 was harvested last. As the population was highly diverse, not all plants were fully matured at harvest. The soil type was Sandy Loam. Fertilisation was done with 1000 kg NPK 14-3-15 per hectare. Pests and diseases were controlled with Fenix and Titus before and right after sprouting (weed), Mospilan in the end of June and again in the end of July (insects), and alternating Ranman and Revus from approximately 23 June and until desiccation as needed, depending on weather (late blight). The fields were irrigated as needed. For evaluation of the robustness of the prediction model, a test panel of 74 individuals (see Online Resource 2) was selected from a mixture of elite cultivars and breeding clones, that have been grown, harvested and phenotyped in the years 1985–2014 in Vandel, Denmark. The cultivars were planted around mid-April to mid-May and harvested in late August and September, where cultivars used for starch production were harvested last. Tubers were desiccated 1–2 weeks before harvesting and plants were generally fully matured at harvest. Otherwise, growing conditions were the same as for the MASPOT panel. Varying amounts of data were available (some years missing) for each cultivar in the test panel. The relationship between the MASPOT population and the test panel is visualised in Fig. 1.

### Phenotyping and adjustment for environmental effects

Dry matter content was determined for offspring harvested late in August 2013 (one replicate) and 2014 (two replicates). The tubers were washed and a basket of 1.5–10 kg of tubers was weighed above and under water, shortly after harvesting. The dry matter content was calculated using the following empirical equation:

$${\text{DM}}\% = 214 \cdot \left( {\left( {\frac{{{\text{weight}}\;{\text{in}}\;{\text{air}}}}{{\left( {{\text{weight}}\;{\text{in}}\;{\text{air}}} \right) - \left( {{\text{weight}}\;{\text{in}}\;{\text{water}}} \right)}}} \right) - 0.988} \right).$$

Starch content was computed from dry matter content as described by the following empirical equation:

$$S\% = {\text{DM}}\% - 5.75.$$

Despite the fact that starch content is not directly determined by the phenotyping method, Behrend et al. (1880) showed that at least for relevant breeding material, this simple equation is sufficiently accurate (Nissen 1967). Furthermore, in our experience, this is used by breeding companies. It may, however, be more correct to address this measure as “starch content estimated from observed dry matter”.

For the test panel, dry matter content was determined for individuals harvested in the years between 1997 and 2014 with varying number of data points for each individual. The starch content was then determined as described above.

Chipping quality was determined as chip colour following frying in oil. From historical data (not shown) this trait is known to be quite robust over different years and phenotyping was done using a single season only (2013). Tubers were stored at 4 °C for approximately two months, after which they were stored at room temperature for 2–6 h prior to frying. 4–6 slices (1–2 mm) of each tuber were then fried in sunflower oil at 180 °C until no more bubbles emerged (typically 2–3 min). Frying colour was assessed visually to a standard set on an arbitrary grading scale from 1 (dark) to 9 (light). Chipping quality for the test panel was assessed in the same way for tubers harvested in the years 1997–2014.

Since data from varying years were used, the phenotypic data were corrected for difference across years by fitting a linear mixed-effects model to the phenotypic data via restricted maximum likelihood (REML) using the following model:

$$y_{ij} = \mu + {\text{genotype}}_{i} + {\text{year}}_{j} + e_{ij} ,$$

where $y_{ij}$ is the observed phenotype, $\mu$ is the overall mean, ${\text{genotype}}_{i}$ is the random effect of the *i*th genotype, ${\text{year}}_{j}$ is the fixed effect of the $j$th year, and $e_{ij}$ is the error term. The model was made with the lme4 package in R (Bates et al. 2014; R Core Team 2015).

### Adapter design

5′ and 3′ barcoding adapters for Illumina sequencing were designed. All adapters contained a 3 bp overhang complementary to the overhang generated by *Ape*KI (CWG), which was the chosen restriction enzyme. In addition, 5′ adapters contained eight different internal 4–8 bp barcode sequences, as described in (Elshire et al. 2011), and 3′ adapters contained 12 different 6 bp barcode sequences compatible with standard Illumina sequencing multiplexing, enabling a 96 multiplexing system. Adapter design ensured that the *Ape*KI recognition site did not occur in any adapter sequence and was not regenerated after ligation to genomic DNA. A list of adapter sequences is given in Online Resource 3.

### Preparation of genotyping-by-sequencing libraries

15–25 mg leaf tissue was homogenised by freezing the sample in liquid nitrogen and subjecting it to 3 × 10 s cycles at 6500 rpm using a Precellys mechanical homogeniser (Bertin Technologies, Montigny le Bretonneux, France). DNA was extracted with DNeasy Plant Mini Kit (QIAGEN, Hilden, Germany) following the manufacturer’s instructions. The resulting DNA samples were digested with *Ape*KI (NEB) and ligated to adapters according to the 96 Plex GBS protocol developed by Rob Elshire (Elshire et al. 2011) with minor revisions. Sets of 96 differently barcoded samples were combined in eight pools and purified using NucleoSpin Extract II kit (Macherey–Nagel, Düren, Germany). Restriction fragments from each library were amplified in 50 µL volumes containing 4 µL pooled DNA fragments using Phusion High-Fidelity PCR kit (Thermo Scientific). Primer design and temperature cycling were performed according to the protocol developed by Elshire (Elshire et al. 2011). Libraries were purified as before and diluted to 2 nM as determined by Qubit (Thermo Scientific). Single-read sequencing (100 bp) was performed on a HiSeq 2000 (Illumina, San Diego, USA). Each 96-plex library was sequenced on three channels of a flow cell. The test panel was prepared in the same manner and sequenced on two rapid run flow cells on a HiSeq 2500 (Illumina, San Diego, USA).

### Filtering raw sequence data, mapping and SNP calling

Sequenced reads were demultiplexed using fastq-multx (Aronesty 2013) sorting the data into separate files, removing the barcode, and discarding reads that did not perfectly match any of the barcodes. Sequencing data were imported into CLC Genomics Workbench v. 7.0 (CLC bio, QIAGEN) and trimmed (minimum quality score 0.01, removal of ambiguous nucleotides and remaining adapter sequences). All processed reads were concatenated to one fastq file per sample, containing trimmed single reads. Reads were mapped to the potato reference genome sequence [DM v4.03 (Sharma et al. 2013)] using BWA and sorted with Picard tools (http://broadinstitute.github.io/picard/). Single-nucleotide polymorphisms (SNPs) and insertions and deletions (INDELs) were called using the Genome Analysis Toolkits (GATK) (McKenna et al. 2010) UnifiedGenotyper tool with the minimum phred-scaled confidence threshold at which variants should be called set to 50, and the minimum threshold at which variants should be emitted (and filtered with LowQual if less than the calling threshold) set to 20. Ploidy was set to 4. SNPs and INDELs were then filtered with a Root Mean Square mapping quality of at least 30, and only biallelic variants were included. Rather than calling genotypes, which would require high coverage sequence reads, the variant allele frequencies at each data point were estimated and used directly in further analysis according to (Ashraf et al. 2014). Minor allele frequency (MAF) was estimated from the read coverage, and SNPs were filtered on a minimum MAF of 1% (average variant allele frequency <0.99 and >0.01). Additionally, SNPs were filtered with a read coverage between 5 and 60 and a maximum number of missing data of 50%. Individuals with greater than 70% missing data were removed.

### Assessment of population structure

All statistical analysis and graphics were performed in R (R Core Team 2015). The relatedness among the individuals was assessed by creating a genomic relationship matrix (${\mathbf{G}}$) from the genotype matrix (${\mathbf{Z}}$) according to VanRaden (2008) with ${\mathbf{Z}}$ containing allele frequencies for each sample and SNP computed from sequence data (Ashraf et al. 2016). The allele frequencies were values between 0 and 1 calculated as the ratio between allele counts of the alternative allele and the total allele count, hence tetraploid allele dosage will also be captured:

$${\text{AF}} = \frac{{{\text{AC}}_{\text{alt}} }}{{{\text{AC}}_{\text{ref}} + {\text{AC}}_{\text{alt}} }}.$$

The allele frequencies were corrected for missing data using the following correction as described by VanRaden (2008, p. 4420):

$$w_{i} = \sqrt {\frac{{\mathop \sum \nolimits p_{k} \left( {1 - p_{k} } \right) {\text{over}}\;{\text{all}}\;{\text{loci}}}}{{\mathop \sum \nolimits p_{k} \left( {1 - p_{k} } \right) {\text{over}}\;{\text{only}}\;{\text{non}} - {\text{missing}}\;{\text{loci}}}}} ,$$

where $p_{k}$ is the mean allele frequency at locus $k$. The genotype matrix was centred and adjusted for missing values as described by Ashraf et al. (2016), after which missing values were set to zero, corresponding to a mean imputation for missing data.

$$\varvec{Z}_{ik} = \left( {\varvec{X}_{ik} - p_{k} } \right) \cdot w_{i} .$$

The genomic relationship matrix was scaled using global scaling (VanRaden method 1) (VanRaden 2008).

$${\mathbf{G}} = \frac{{{\mathbf{Z}}^{\prime } {\mathbf{Z}}}}{{0.25\sum {p_{k} } \left( {1 - p_{k} } \right)}},$$

where $0.25\mathop \sum \nolimits p_{k} \left( {1 - p_{k} } \right)$ is the sum of genotype variance and also the average diagonal of ${\mathbf{Z}}^{\prime } {\mathbf{Z}}$.

A principal component analysis (PCA) was also performed on the genotype data to detect population structure.

### Heritability

The heritability of each trait within the MASPOT population was estimated from both pedigree and genomic data. The pedigree heritability was estimated as the slope of the regression line between parent and offspring phenotypic data. Parent phenotypic data were calculated as the mean of the mother and father phenotypic data. Offspring phenotypic data were calculated as the mean over all offspring to each parent pair. The genomic heritability was estimated as the ratio of the genomic and the phenotypic variance, where the genomic variance is obtained with a REML analysis using the genomic relationship matrix (de los Campos et al. 2015).

$$h^{2} = \frac{{\sigma_{g}^{2} }}{{\sigma_{y}^{2} }}.$$

### Statistical models

Three different statistical models were used to estimate GEBVs: GBLUP, BayesA, and BayesC. The GBLUP method uses the genomic relationship matrix, and it is equivalent with a ridge-regression model with uniform shrinkage of SNP effects regardless of the marker effect size, although shrinkage is dependent on sample size and allele frequency (Gianola 2013). It is the most common used parametric method for GS. GBLUP directly estimates genomic breeding values with the model (Meuwissen et al. 2001).

$$\varvec{y} = 1\mu + \varvec{g} + \varvec{e},$$

where $\varvec{y}$ is a vector of phenotypes, $\mu$ is the mean, $\varvec{e}$ is a vector of random normal deviates, and $\varvec{g}$ is a vector of random genomic breeding values with the distribution:

$$\varvec{g}\sim N\left( {0,\varvec{G}\sigma_{g}^{2} } \right).$$

The Bayesian models, however, allow the markers to explain different amounts of variation. In BayesA, each marker effect is drawn from a normal distribution with its own variance, allowing the marker to be shrunken toward zero to a different degree (Meuwissen et al. 2001).

$$\varvec{y} = 1\mu + \varvec{Xb} + \varvec{e},$$

where $\varvec{X}$ is the design matrix of all marker effects and $\varvec{b}$ is a vector of marker effects. Each marker effect is assumed to have its own variance parameter:

$$b_{i} \sim N\left( {0,\sigma_{bi}^{2} } \right),$$

and where the prior distribution for all variances is a scaled inverted Chi-square distribution:

$$\sigma_{bi}^{2} \sim \chi^{ - 2} \left( {\upsilon ,S} \right),$$

where $\upsilon$ is the number of degrees of freedom and $S$ is a scale parameter.

BayesC assumes the marker effects to be a mixture, with most marker effects to be zero, and a (usually) smaller part of markers to be nonzero. There is a common marker effect variance for all markers with nonzero effect.

$$b = \left\{ {\begin{array}{*{20}l} 0 \\ {\sim N\left( {0,\sigma_{b }^{2} } \right).} \\ \end{array} } \right.$$

All models were fitted with the BGLR package in R (de los Campos and Perez Rodriguez 2016) with default settings for priors. 12,000 iterations were used and a burn-in setting of 2000. All analyses within the MASPOT population were performed using eightfold cross-validation schemes. The data were randomly divided into eight groups and one group was then used as validation set while the remaining seven groups were used as training population. The process was repeated, each time with another group as validation set, until predictions had been obtained for all individuals. Each analysis was repeated with 10 different cross-validation groupings and the average GEBV over the ten samplings was taken. A leave-sibs-out cross-validation scheme was also applied to analyses within the MASPOT population, in which individuals were split into groups of full- and half-sibs. Essentially, the 18 parents used for the MASPOT population were split into nine pairs, and the offspring were then divided into nine groups based on the parents, such that each group contained all offspring to one or both parents of the pair in question. Predictions were performed for every group, while making sure that full- and half-sibs were not present in both the training population and the validation population simultaneously. Most individuals were present in two groups, and thus present in the validation population twice, and in which case, the average GEBV was calculated for further analysis. The accuracy of the GEBVs was determined as the Pearson correlation between the GEBVs and the observed phenotypes, described in this paper as prediction correlation:

$$r\left( {\varvec{GEBV}:\varvec{y}} \right).$$

### Assessment of robustness of predictive model in a test data set

The robustness of the prediction models was also assessed by calculating GEBVs of 74 individuals in the test panel and comparing to phenotypic data. Models were created using the MASPOT population and predictions were then made on the test panel with 10 replications. Prediction correlations were calculated as described above. In addition, a combined model was made, using both the MASPOT population and the test panel. Random eightfold cross-validation schemes were made as before, dividing all individuals in eight randomly selected groups of equal sizes. Each analysis was repeated with ten different cross-validation groupings and the average GEBV over the ten samplings was taken.

### Marker selection with GWAS

A GWAS was performed within the MASPOT population on the GBS data by fitting the phenotypic data to each SNP with the genomic relationship matrix as covariance. The basic GBLUP model described before was used and marker effects ($\beta$) were added one at a time:

$$\varvec{y} = 1\mu + \varvec{x}\beta + \varvec{g} + \varvec{e},$$

where $\varvec{x}$ is an $n \times 1$ marker genotype vector for $n$ individuals at a marker locus and $\beta$ is the marker effect. GWAS was performed with the regress package in R (Clifford and McCullagh 2006, 2014). Significance thresholds for p-values were determined for each chromosome as the false discovery rate (FDR) as described by Magwene et al. (2011). New genomic relationship matrices were made using only the significant SNPs and predictions were then made in the test panel as described above.

## Results

### Genotypes and assessment of population structure

The sequencing of the MASPOT population yielded on average 4 million trimmed and filtered reads per sample for the MASPOT population and 1.5 million trimmed and filtered reads per sample for the test panel. A total of 3.4 million variant sites were found. Following filtering for MAF >1% estimated from read coverage, maximum missing data of 50%, and a minimum SNP coverage of 1, 505,321 SNPs remained. Filtering for minimum coverage of 5 reduced SNPs further to 186,757 SNPs, and finally, removing SNPs with coverage above 60 gave 171,859 SNPs, constituting the marker set used (see Online Resource 4). 18 samples that contained less than 30% of the selected SNPs were removed, resulting in 755 samples remaining in the MASPOT population and 63 samples in the test panel. Markers were well-distributed across the chromosomes with the highest marker density in high gene density regions typically found in the ends of chromosomes, while it was lowest in highly repetitive centromeric regions (see Fig. S1 in Online Resource 5). In fact, in many organisms, crossing over and gene conversion seems to be repressed near centromeres (Resnick 1987; Sherman and Stack 1995; Talbert and Henikoff 2010) resulting in less genetic diversity, which explains the low marker density in those regions. This is similar to what has been observed in similar studies in other crops (Ganal et al. 2011; Sonah et al. 2013).

No clear population structure was detected from the heat map of the genomic relationship matrix (Fig. 1). This was further confirmed by a principal component analysis of the genotype data, as the first three principal components explained only 3.8, 2.9, and 2.7% of the total variation, respectively (Fig. S2 in Online Resource 5), which are indeed typical values for data with family structure, but no substantial population structure (Arruda et al. 2015). Due to the diallel design of the MASPOT population from 18 parents, family structure was expected, and this is detected in Fig. 1, where clusters around the diagonal indicate strong genetic relationships between full and half sibs. However, the lack of stronger relationships across the entire population is evident to the highly diverse and heterozygous nature of tetraploid potato. Values between −0.22 and 0.96 were observed in the genomic relationship matrix for the MASPOT population, yet 70% of the values were below 0, reflecting individuals that are less related than average, contrary to positive values that reflect individuals that are more related than average (VanRaden 2008). No strong relationships were detected between individuals in the test panel and the MASPOT population, while some relatedness could be observed within the test panel. In addition, the genetic diversity within the test panel was quite low, as evident from the PCA plot (Fig. S2 in Online Resource 5), contrary to the MASPOT population. The PCA plot also revealed clustering of full-sibs. Furthermore, individuals clustered in high and low starch content, forming a gradient from low to high values (see Fig. S3 in Online Resource 5), but interestingly, the same was not true for chipping quality.

### Phenotypes and heritability of starch content and chipping quality

**Fig. 2:**
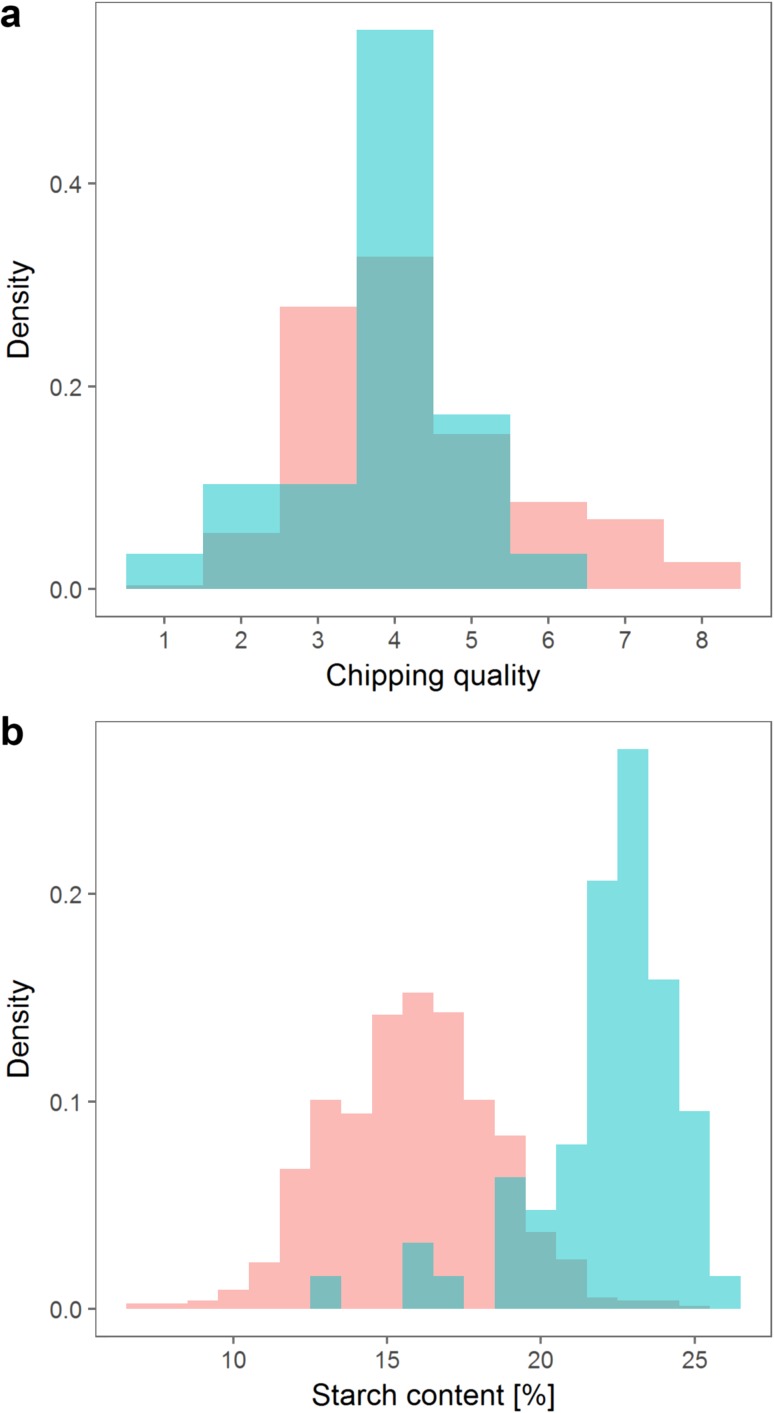
Density histograms depicting the phenotype distributions for the MASPOT population (*red*) and the test panel (*blue*). Chipping quality **a** was determined as assessment of *frying colour* on a scale from 1 (poor) to 9 (best), while starch content **b** was measured as percentage

Chipping quality was evaluated on a scale from 1 to 9. After correcting the phenotypic data for differences between years, the MASPOT population covered the scale from 0.6 to 7.6, slightly skewed towards the lower end (Fig. 2). The test panel chipping quality data were normally distributed with a similar mean as the MASPOT population but a narrower range from 0.8 to 5.8. Chipping quality data were only available for 69% of the MASPOT population and 48% of the test panel.

The phenotypic distribution of starch content was normally distributed for the MASPOT population, ranging from 6.5 to 24.8% (Fig. 2). The test panel had a quite different distribution, with a narrower range and a mean of 22%, similar to the maximum phenotype in the MASPOT population. Three individuals in the test panel lie slightly outside the range of the MASPOT population. However, even though this is in principle a violation of model usability, removing the three individuals did not seem to have any effect on the prediction models (data not shown), and it was thus determined that it does not seriously affect the usability of the models. The phenotypic distributions reflected the composition of the MASPOT population versus the test panel; the test panel consisted of a selection of varieties and advanced breeding clones that have been selected for several traits, including starch content and chipping quality, whereas the MASPOT population had not been subjected to any selection.

**Fig. 3:**
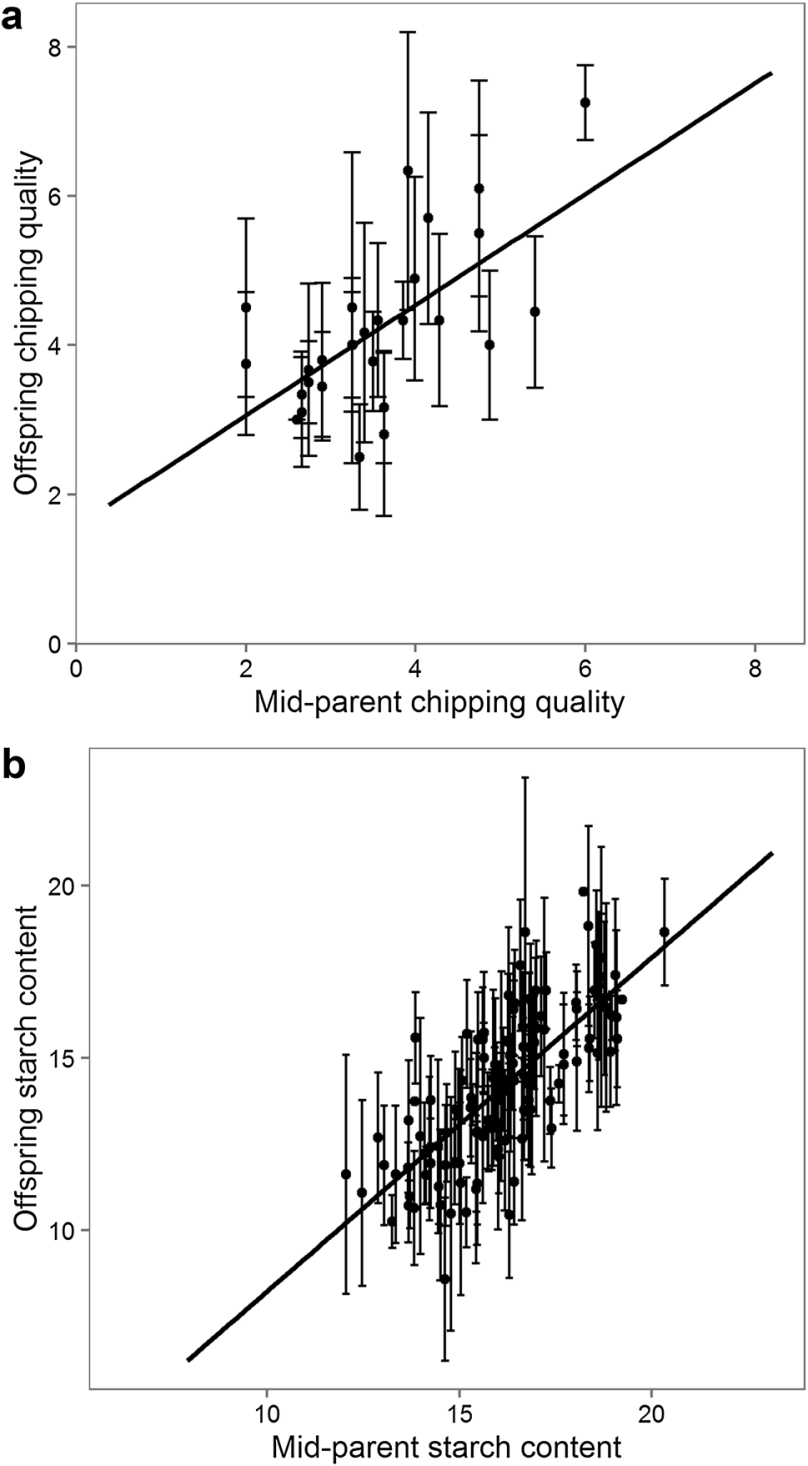
Parent-offspring regressions for the offspring in the MASPOT population for chipping quality (**a**) and starch content (**b**) for estimating pedigree heritabilities. The average phenotypic value of each parent pair is plotted versus the average offspring value. *Error bars* depict the standard deviation. Narrow sense heritability is estimated as the slope of the curves

Two estimates of narrow sense heritability for the MASPOT population were made. First, the heritability was estimated as the ratio between observed genomic and observed phenotypic variance. Second, heritability was estimated from parent-offspring regression (Fig. 3) using phenotypic data only. For starch content, the estimated heritability differed markedly by the two methods; 41% estimated from genomic data, and 90% estimated from parent-offspring regression. For chipping quality, the heritability estimated from the two methods was more similar, ranging from 65% for the genomic heritability to 78% for the pedigree-estimated heritability.

### Marker selection with GWAS

372 SNPs were found to be significantly associated with the chipping quality trait, while 612 SNPs were found significant for starch content. The SNPs were mainly found on chromosome 10 for both traits, namely 316 SNPs for chipping quality and 596 SNPs for starch content, and 234 of those were found significant for both traits. The GWAS results can be seen in Fig. S4 in Online Resource 5, and all significant SNPs are listed in Online Resources 6 and 7.

### Predictive models within the MASPOT population

**Fig. 4:**
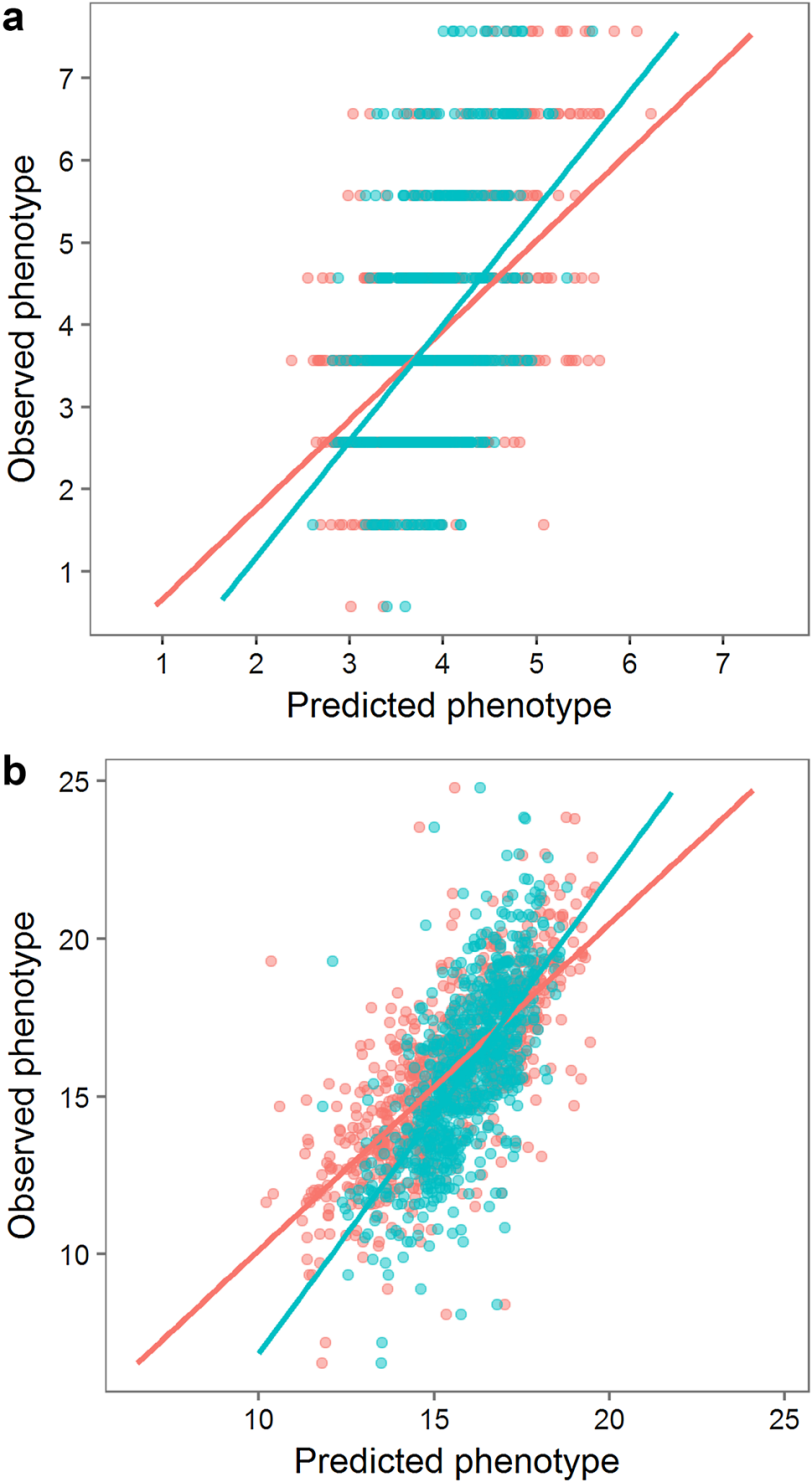
Distribution of genomic estimated breeding values and observed phenotype values for BayesC model for the MASPOT population for chipping quality (**a**) and starch content (**b**). *Red colour* indicates predictions made with eightfold cross-validation. *Blue colour* indicates predictions made with leave-sibs-out cross-validation. The slopes of the regression lines indicate the degree of bias of the predictions

The genotypes were regressed to the phenotypes of the MASPOT population and GEBVs were estimated for each individual using three different prediction algorithms: GBLUP (VanRaden 2008), BayesA (Meuwissen et al. 2001), and BayesC (Habier et al. 2011). Prediction correlations were quantified within training data using either eightfold cross-validation schemes repeated 10 times or a leave-sibs-out cross-validation scheme. The three prediction algorithms yielded similar prediction accuracies for each trait (Table 1). Prediction correlations estimated with eightfold cross-validation were 0.56 for chipping quality and 0.73 for starch content. The predictions were unbiased, estimated from the slope of the regression line between the observed (*y*) and the averaged predicted values (*x*) (Fig. 4), where *β* of 1 indicates no bias. When using the leave-sibs-out cross-validation scheme, prediction correlations were slightly lower while bias was larger.

**Table 1:** Prediction correlations and bias found within the MASPOT population from average GEBVs over 10 repeats with BayesA, BayesC, and GBLUP

Trait/Cross validation	BayesA		BayesC		GBLUP	
	Correlation	Bias	Correlation	Bias	Correlation	Bias
Chipping quality [524]						
8-fold	0.56	1.07	0.56	1.09	0.56	1.10
Leave-sibs-out	0.47	1.34	0.47	1.41	0.47	1.41
Starch content [755]						
8-fold	0.73	1.02	0.73	1.03	0.73	1.04
Leave-sibs-out	0.67	1.43	0.68	1.51	0.68	1.50

8-fold random cross-validation and leave-sibs-out cross-validation systems were used for each case

The number of phenotypes available for each trait is indicated with square brackets

### Assessment of predictive power in an unrelated population

**Fig. 5:**
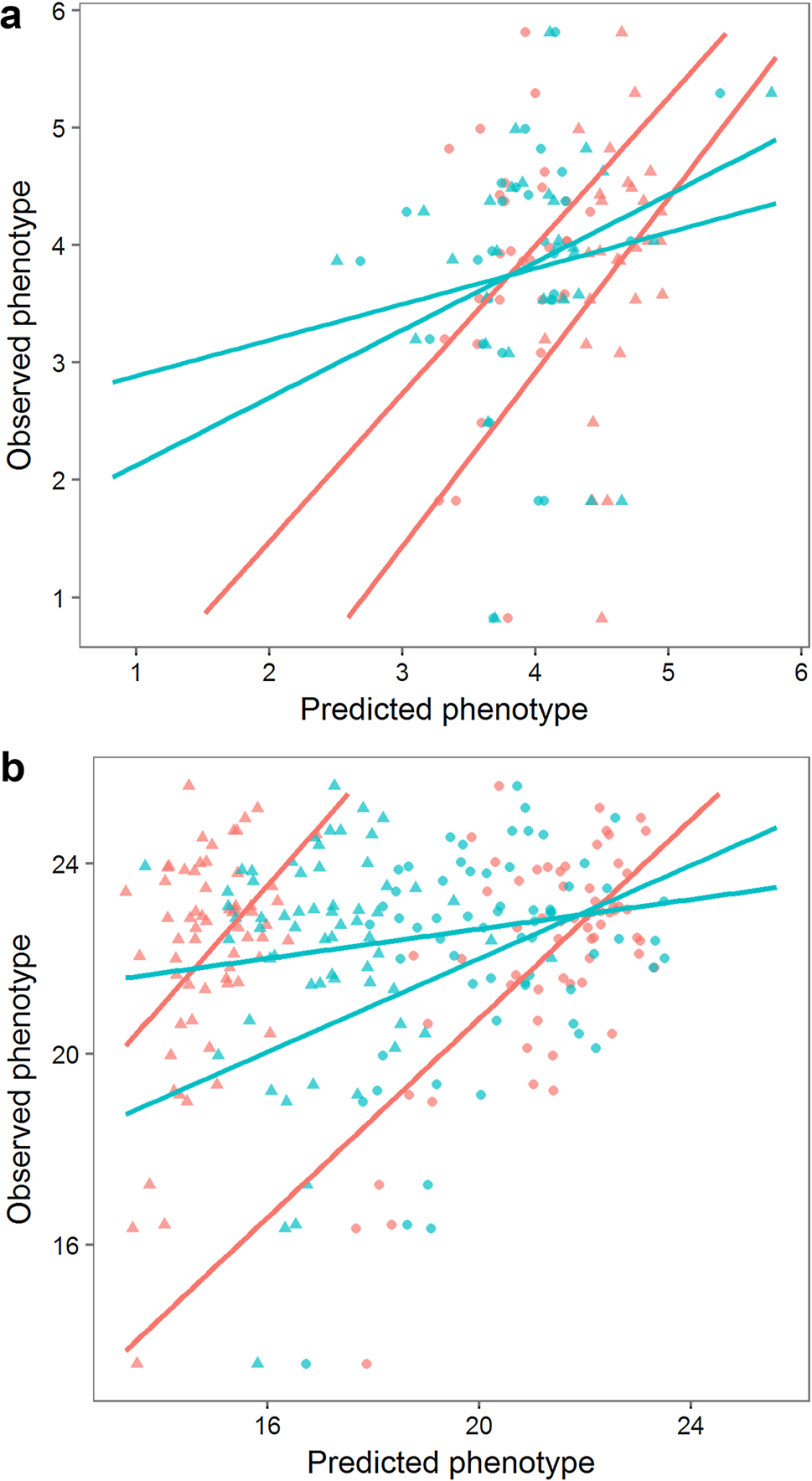
Distribution of genomic estimated breeding values and observed phenotype values for BayesC model for the test panel for chipping quality (**a**) and starch content (**b**). *Red colour* indicates predictions made with all 171,859 SNPs. *Blue colour* indicates predictions made with GWAS selected SNPs. *Triangle* indicates model trained with the MASPOT population. *Circle* indicates model trained with the combined set (MASPOT population and test panel) with eightfold cross-validation. The slopes of the regression lines indicate the degree of bias of the predictions

Since the purpose of predictive models is to estimate GEBVs in individuals outside the training population, we tested the ability of our model to predict starch content and chipping quality in an unrelated test panel of potato lines. As mentioned before, the test panel consisted of varieties that have been selected for several traits, including starch content, and particularly the mean phenotype of starch content was at the high end. This represents a challenging, but not unrealistic case for testing the robustness of the predictive model. Prediction correlations between 0.30 and 0.31 were observed for chipping quality and 0.42 and 0.43 for starch content (Table 2; Fig. 5). Compared to predictions within the MASPOT population, the prediction bias was large for the test panel, being 1.34–1.48 and 1.24–1.26 for chipping quality and starch content, respectively, hence the highest scores were underestimated, while the lowest were overestimated.

**Table 2:** Prediction correlations and bias found from average GEBVs over 10 repeats with BayesA, BayesC, and GBLUP

Trait/test set	Training set	Markers	BayesA		BayesC		GBLUP	
			Correlation	Bias	Correlation	Bias	Correlation	Bias
Chipping quality								
Test panel [30]	MASPOT	All [171,859 SNPs]	0.31	1.34	0.31	1.48	0.30	1.47
Test panel [30]	MASPOT	GWAS [372 SNPs]	0.16	0.29	0.17	0.31	0.17	0.29
Test panel [30]	Combined^*^	All [171,859 SNPs]	0.36	1.21	0.36	1.26	0.37	1.32
Test panel [30]	Combined^*^	GWAS [372 SNPs]	0.28	0.61	0.26	0.58	0.30	0.63
MASPOT [524]	Combined^*^	All [171,859 SNPs]	0.56	1.07	0.56	1.09	0.56	1.09
Combined [554]	Combined^*^	All [171,859 SNPs]	0.56	1.07	0.55	1.09	0.55	1.09
Starch content								
Test panel [63]	MASPOT	All [171,859 SNPs]	0.43	1.24	0.43	1.26	0.42	1.26
Test panel [63]	MASPOT	GWAS [612 SNPs]	0.09	0.16	0.09	0.15	0.11	0.19
Test panel [63]	Combined^*^	All [171,859 SNPs]	0.65	1.04	0.65	1.04	0.65	1.04
Test panel [63]	Combined^*^	GWAS [612 SNPs]	0.34	0.49	0.34	0.49	0.34	0.48
MASPOT [755]	Combined^*^	All [171,859 SNPs]	0.73	0.99	0.73	1.00	0.73	1.00
Combined [818]	Combined^*^	All [171,859 SNPs]	0.81	1.05	0.81	1.05	0.81	1.06

Predictions were made using either only the MASPOT population or both the MASPOT population and the test panel (combined) to train the model. Predictions of the test panel were also performed using only the significant SNPs selected with GWAS. Predictions made with the combined model were done using eightfold cross-validation (*)

The number of phenotypes available in each case is indicated with square brackets

For predictions made using only the significant SNPs selected with GWAS, prediction correlations were considerably lower, or 0.16–0.17 for chipping quality and 0.09–0.11 for starch content. Large biases were also observed, however, contrary to when using all SNPs for prediction, the slopes were below one, giving the opposite situation from before.

### Assessment of predictive power in an expanded training population

To estimate the value of expanding the model, a combined model was made, where both the MASPOT population and the test panel were used as a training population to create a predictive model. 10 different eightfold cross-validation systems were made and GEBVs were calculated. Prediction correlations were calculated for the two panels separately and for the combined panel. For starch content, prediction correlations of 0.65 were obtained from average GEBVs for the test panel (Table 2), while correlations ranged from 0.62 to 0.67 for the ten different cross-validation sets. When using the GWAS selected SNPs for prediction, lower prediction correlations were obtained at 0.34 and a large bias of 0.48–0.49. Prediction correlations for the MASPOT panel using the expanded training population were similar to when using only the MASPOT population for the modelling. Prediction correlation for the combined set was higher than the prediction correlation for the MASPOT population, with an average of 0.81, despite that the same training set (combined) was used in both.

For chipping quality, there was a considerable variation in the prediction correlations and bias for the test panel for each cross-validation set. Prediction correlations ranging between 0.15 and 0.48 were obtained while the bias varied from 0.54 to 1.72. Prediction correlation of average GEBVs was 0.36–0.37 with a bias of 1.21–1.32. There was a correlation between the prediction correlations and the bias, where the higher the prediction correlation, the larger the bias. Predictions with GWAS selected SNPs resulted in slightly lower prediction correlations for the test panel and a substantially larger bias (0.26–0.28 and 0.58–0.63). No change was seen in prediction correlation for the MASPOT population when using the expanded training population compared to using only the MASPOT population for modelling.

## Discussion

### Population

The MASPOT population in this study was a selection of entries from a larger population made from a full diallel crossing design. The crosses were generated from 18 tetraploid cultivars and breeding clones that were selected to be as unrelated as possible in the inbred elite potato germplasm to create as diverse offspring as possible. The variation in genotypes was thus vast in this population, which is an advantage when creating prediction models of this type. Family structure was detected as strong relatedness between full- and half-sibs in both the heat map and the PCA plot. In addition, the high heritability of starch content was evident from the clustering in the PCA plot, as there was a clear clustering of families into high and low starch content. Interestingly, although a high heritability was also estimated for chipping quality, the same pattern was not seen in the PCA plot, but in contrast, there seemed to be a more random distribution of high and low chipping quality values among full-sibs. Granted, this can simply be an effect of the lower number of individuals with available chipping quality data compared to starch content, thus there were fewer individuals from each family and the clustering effect was, therefore, not as obvious. No clear population structure was detected, indicating that the genetic diversity between alleles is the dominating component, regardless of whether they are found within a single parent or between parents. The parents each represent a relatively random selection of four alleles from the gene pool and not a particular set of four distinct from other parent allele sets. Indeed, the fact that in such a population only weak population structure was observed is testament to the extreme haplotype diversity of potato. From resequencing 400 bp amplicons of exons each containing multiple SNP loci of a panel of 48 potato varieties, we obtain an average of 11 haplotypes per locus (data not shown). From genome sequencing of potato and tomato, the nucleotide divergence of the elite population of potato is estimated to be six-fold higher (3.5% SNP positions in the heterozygous RH variant) than the divergence of domesticated tomato (0.6%) from the wild species *Solanum pimpinellifolium* (Potato Genome Sequencing Consortium 2011; Tomato Genome Consortium 2012). This diversity dominated over population and family structure in the SNP data. Alternatively, the absence of population structure could in principle be caused by an extremely narrow genetic base of the 18 parents, so that they were all derived from the same common recent genetic ancestor. However, the observed genetic diversity is not in agreement with this explanation.

### Heritability

The heritability of each trait was estimated with two methods; from pedigree data and from genetic data. For chipping quality, the heritability was similar for both methods. For starch content, however, there was a substantial difference between those two heritabilities, ranging from 40% for the genomic heritability to 90% for the pedigree heritability. The genomic heritability can be defined as the proportion of variance of phenotypes explained by the regression on available markers. Many polymorphic markers are needed to estimate relatedness accurately, in particular for distant relatives. Given the high diversity of potato with one SNP per 24 bp (Uitdewilligen et al. 2013), and an average of 11 haplotypes per loci observed in only 48 individuals (data not shown), the true genomic diversity is likely to be underestimated using only 171,859 markers. Each biallelic SNP marker is likely to be in linkage with more than two haplotypes, and thus cannot represent the true diversity at the site, hence the heritability of 90% observed from the parent offspring regression is the more credible of the two. This extremely high heritability estimate, however, is not in agreement with other studies. For instance, Slater et al. (2014b) estimated the heritability of specific gravity, which is highly related to our measure of starch content, to 74%. In contrast, D’hoop et al. (2014) estimated heritabilities of underwater weight, related to starch content, to 87% for one panel, while another panel was estimated to have heritability of 76%. As heritability is a function of genetic and environmental variances, it is thus appropriate only to the population from which the variances are derived (Cunningham and Stevenson 1963). In this case, the genetic diversity is vast due to the diallel crossing design and the fact that no selection has occurred in the MASPOT population, and thus high observed heritabilities can be expected.

### GWAS

SNPs that were determined to be significant for either starch content or chipping quality with GWAS were mainly clustered on chromosome 10. In a recent study of GWAS in tetraploid potato, Rosyara et al. (2016) did not identify any QTL for complex traits such as chip colour, however, this is most likely due to insufficient markers and population size (3441 SNP markers and 221 tetraploid lines). A number of invertases believed to be associated with a range of tuber quality traits are located on chromosome 10 (Schreiber et al. 2014), including apoplastic invertase (*Inv*-*ap*-*b*) (Li et al. 2008). However, the GBS data set used in this study is not well suited for the discovery of exact genetic variants of genes underpinning observed functional biological difference. This would require a different SNP discovery approach altogether, or at least a deeper sequencing of each sample to ensure less observed markers to be filtered out or result in false homozygous calls (see below) to expect a realistic chance of efficiently identifying causal variation. As a consequence, we have not conducted such an investigation.

### Genomic prediction models

In this study, genomic prediction models were developed with GBLUP, BayesA, and BayesC to predict phenotypic values. GBLUP mostly assumes equal variance across all loci and thus performs homogeneous shrinkage of marker effects, although unequal shrinkage can occur at extreme MAF (Gianola 2013). The Bayesian models, however, allow the marker loci to explain different amounts of variation and they are therefore more flexible with respect to genetic architecture. Compared to GBLUP, Bayesian methods are thus better suited for traits controlled by few large-effect QTL (Meuwissen et al. 2001; Clark et al. 2011; Beaulieu et al. 2014). Starch content and chipping quality are considered to be highly polygenic traits (van Eck 2007), and the difference between model performance were thus not expected to be considerable. Indeed, the three models performed similarly.

Prediction models within the MASPOT population were made with either a random eightfold cross-validation set or a leave-sibs-out cross-validation system. For both traits, the prediction correlations obtained with the leave-sibs-out cross-validation were lower than those obtained with the eightfold cross-validation as expected. Random cross-validation can lead to overestimation of the actual performance in breeding, because full-sibs and half-sibs performance are easier to predict than less related individuals, which are likely to be the target of predictions in practical breeding. However, a disadvantage with the leave-sibs-out cross-validation scheme given the high genetic diversity of potato in relation to the size of the training population, is that this method is likely to exclude relevant haplotypes with functional importance from the model altogether, because any particular haplotype is not necessarily present outside siblings. To ensure this, a larger training population is necessary. At the present time in the absence of larger data sets, we cannot establish which of the two cross-validation schemes is the more realistic.

### Validation of models in the test panel

Moderate prediction correlations were obtained when predicting chipping quality in the test panel, although prediction bias was large. Also for starch content, moderate prediction correlations were obtained in the test panel, considerably lower than the correlations obtained within the MASPOT population, most likely due to the profound differences between the two panels. The individuals in the test panel have been selected for high starch content, while the MASPOT population has not been subjected to any selection. This was clearly reflected in the phenotypes, where average starch content was much different between the two populations. Additionally, the high genetic diversity of potato means the validation panel likely contains haplotypes of moderate to high effect size not accounted for in the MASPOT population. The logical solution is to increase the size of the training population to match the high genetic diversity. When we combined the two populations, we obtained higher and unbiased prediction correlations without impacting the prediction accuracy of the MASPOT population.

It should also be considered, though, that the test panel consisted of historical data from the years 1997–2014, while phenotype data for the MASPOT population was collected in 2013 and 2014. The use of a prediction model constructed from contemporary data to predict a panel with historical data might have a negative effect on the prediction accuracy due to imprecise pre-processing of the phenotypic data to adjust for annual environmental variation. However, studies in barley (Sallam et al. 2015) and oat (Asoro et al. 2011) have shown that only a slight decrease in accuracy was observed when using historic data to train a prediction model compared to contemporary data. In addition, Sallam et al. (2015) noted that the historical data used in the study on barley were unbalanced and not corrected for field variability, contrary to the contemporary data, suggesting that the fact that the data were collected in another time period was not the only factor affecting the prediction accuracy. In the present study, the phenotypic data has been corrected for yearly variation. If data from the validation panel from 2013 and 2014 only were used (omitting 20 individuals from the validation panel for which there was no 2013–2014 data) slightly improved prediction correlation of 0.50 was observed, but also a higher bias of 2.0. The correlation, however, was still substantially lower than the 0.74 observed for the MASPOT population, suggesting that imprecise pre-processing of phenotype data cannot fully explain the observed difference in accuracy between the two populations. The data was, however, not corrected for genotype-by-environment (GxE) interaction. Generally, little is known about specific GxE interaction in potato and our data does not allow a rigorous estimate of GxE effects. Indications from incomplete data of potato cultivars grown in various locations over many years generally display the same ranking of cultivars with respect to starch content and chipping quality from year to year, at least within the same climate zones. This indicates a limited importance of GxE interactions in this study. Thus, for simplicity, and to decrease the risk of overfitting the data, we have chosen to ignore GxE in our data correction.

Predictions for chipping quality were more variable when using the combined populations to construct the models. For ten different eightfold cross-validation sets, prediction correlations ranging from 0.15 to 0.48 were obtained, with bias ranging from 0.54 to 1.72. To this end, it is important to note that the test panel only consisted of 63 individuals, and of those, only 30 individuals had available chipping quality data. It could be argued that the number of individuals with chipping quality phenotype data was too low to provide reliable estimates of correlation because of insufficient degrees of freedom. Nonetheless, this number of living individuals (excluding full and half sibs) with a comprehensive set of phenotype data is a situation often encountered in practical breeding and is thus relevant for this study. Indeed, the 30 individuals with these phenotype data were all that was available at the commercial breeding company LKF Vandel.

As argued above but even more relevant for chipping quality, it is likely that every subgrouping of the panel leads to the exclusion of moderate to high effect size haplotypes from the model. Considering the complexity and gene redundancy (gene copy number) of the carbohydrate metabolism in potato, it is not difficult to imagine that the extent of reducing sugar accumulation following cold storage can be influenced by many haplotypes and that for reliable chipping quality prediction across the spectrum of elite varieties thus requires a relatively large training population. However, in contrast to starch content, combining the MASPOT population and the test panel did not improve prediction accuracy for chipping quality markedly as expected. This could simply stem from the design of the cross-validation system, since the lower number of individuals with phenotype data available in the test panel for chipping quality compared to starch content (30 versus 63) would not be equally divided between the cross-validation groups and in some folds, the majority of test panel individuals might be in the validation set by chance. However, if this effect was a major contributor, then an inverse correlation between number of test panel individuals in the validation set and prediction correlation should occur. To this end, we have plotted the prediction correlation and number of test panel individuals in Fig. S5 (Online Resource 5), and we observed no systematic distribution of points indicating an inverse relationship. Therefore, we conclude that this is not a major contributor of the marginally increased prediction correlation obtained with the combined model. Another possible explanation for this oddity is that one or more of the high effect size haplotypes does not display additive genetics. Indeed, this may very well be the case for chipping quality. Invertases have been shown to be important for chipping quality (Baldwin et al. 2011; Schreiber et al. 2014) and more than 20 loci encoding invertases exist in potato (Schreiber et al. 2014). While vacuolar invertase have been found to be particularly important for this trait (Sowokinos 2001), and indeed knock-out of this locus leads to high chipping quality (Clasen et al. 2016), this was based on analysis of only four cultivars of potato. It must be considered possible, that good chipping quality can be influenced by other invertase loci. If the case is that multiple invertase loci individually can reduce the concentration of reducing sugars sufficiently to obtain high chipping quality, the effect of these loci is not additive, as each of them will have full effect. In any case, more detailed studies are necessary to support these speculations.

Using GWAS selected SNPs did not improve prediction accuracy when predicting the performance of individuals in the test panel. In fact, prediction correlations of chipping quality were reduced by half, while prediction correlations of starch content were reduced by more than four times when using SNPs specifically selected to be significant for the trait. When using the combined model, predictions were somewhat improved compared to using only the MASPOT population for training, but in all cases, the bias was considerably large. This observation of higher robustness of “cross-breed” prediction may be considered an argument in favour of using numerous genome-wide markers as in GS in comparison with limiting the prediction models to only the most important SNPs as in GWAS. This indicates that GS is a promising alternative to MAS in breeding programmes. While the clear advantage of MAS is that fewer markers need to be sequenced, GS seems to be more robust when predicting performance in unrelated populations, which is a relevant scenario for breeding companies. It is possible to imagine that predictions with GWAS-selected markers could have been improved by conducting GWAS for a reference population that is closely related to the test panel, and applying the identified SNPs in GS on the test panel. The magnitude of the effect of any particular SNP is likely to differ between populations and using GWAS, SNPs that have a smaller effect (below the significance criteria) but larger effect in the testing population are excluded from the model altogether. However, this would require a constant calibration of the prediction model. In addition, the genome-wide prediction approach has the clear advantage that the same set of markers can be used for multiple traits, while the marker selection approach requires separate marker set for each trait.

### Implementation of genomic selection in potato breeding

Overall, the results imply that GS has great potential in predicting the performance of tetraploid potato. The predictions were most accurate when preditcting performance within the same population (MASPOT population), while it loses some power when predicting the performance of unrelated clones. This suggests that GS could initially be most cost-effectively implemented in a potato breeding programme as selection of parents. Rather than attempting to predict performance in unrelated populations, a GS breeding programme could be established by assembling a training population of potential parents and cultivars that are relevant to the breeding programme. Selection could then be made on which cultivars could be used as parents to create the next breeding population using in silico breeding simulations.

Due to the high allelic diversity of potato it will be necessary to construct large training populations that include entries covering the range of a trait’s variance. This contrasts with the traditional habits of the breeding companies, where poorly performing individuals are eliminated from the program at an early stage and before robust phenotyping has been undertaken. Using such truncated data as training sets will not lead to a precise estimation of both positively and negatively acting haplotypes.

A challenge with GBS when expanding the training population is that the number of markers meeting the frequency thresholds to be included in the models decreases. This is a result of undersampling of the distribution of DNA fragments derived in the GBS procedure. Thus, for large training populations it may be necessary to increase sequence depth and/or include imputation algorithms. However, since a key feature of GS is that markers covering the whole genome would potentially explain all genetic variation (Meuwissen et al. 2001; Goddard and Hayes 2007), higher marker density might not be necessary to increase prediction accuracy. Thus, high prediction accuracies can be obtained if marker coverage is sufficient and markers are in linkage disequilibrium with QTL. In fact, previous studies in other species reported high prediction accuracies with much lower marker numbers (Lorenzana and Bernardo 2009; Heffner et al. 2011; Arruda et al. 2015; Slater et al. 2016). In wheat, for example, Arruda et al. (2015) found that appropriate marker coverage was obtained with 1500 to 3000 SNPs, depending on the trait, and including more markers only led to diminishing gains in prediction accuracy. Furthermore, Muir (2007) showed that when the marker coverage of the genome was sufficient, the size of the training population became more important in increasing prediction accuracy, and that increasing the marker density without a corresponding increase in population size can actually decrease prediction accuracy due to increase of collinearity between markers, which has been found to produce overfitted models.

A more fundamental problem for potato is that the prediction models in this study were based on SNPs. At any given SNP position only two values can be obtained (when using biallelic variants only), and since in contrast to most other species where GS has been implemented, more than two haplotypes exist at each locus, a SNP is most likely to represent groups of haplotypes. Such a group is very likely to contain haplotypes of varying effect size and only occasionally be linked uniquely to a specific haplotype. Indeed, this problem may be the reason why MAS has been so inefficient in potato. The markers employed (SNPs, SSR, etc.) have not faithfully represented the true haplotype diversity, and following crossing and segregation of the group of haplotypes linked to the molecular markers, each of these haplotypes would retain linkage to the molecular marker, but they would have different effect size, and thus the desired linkage to the phenotype is lost. Nonetheless, in this study, using a relatively high number of SNPs, we have obtained quite good prediction power. However, increasing the information content of the molecular markers obtained by GBS using the combinations of SNPs found in single reads as haplotype signatures may very well increase prediction accuracy because a higher degree of unique linkage between the molecular markers and the relevant true haplotype structure is obtained. Some information regarding haplotype phasing of SNPs could be captured by counting entire sequence reads instead of single SNPs. This would, however, require the development of an entirely new bioinformatic work flow and is likely to demand high coverage of each GBS site to capture the information, adding to the cost of genotyping, and this was beyond the scope of this study.

Using GBS as genotyping technology comes with the advantage of cost efficiency and the possibility of SNP discovery and detection simultaneously. However, disadvantages are also part of the technology. The presence/absence of a particular restriction enzyme marker site across alleles and low sequence coverage can lead to erroneous estimates of zygosity and results in that a considerable number of haplotypes are not detected (Poland and Rife 2012; Heslot et al. 2013; He et al. 2014; Huang et al. 2014). This is mostly important when using GBS as a causal SNP discovery tool, which we do not attempt to do. In genomic selection, this potential error is overcome by employing a very high number of markers. Hence, even though one marker site, theoretically in highest LD with the phenotype, is influenced by the described effects, closely located adjacent sites would be in LD with the phenotype and will be used in the prediction models instead (Meuwissen et al. 2001; Goddard and Hayes 2007).

GS is still in its infancy within plant breeding, and one of the biggest obstacles for implementing GS in practical breeding is the high start-up costs required. The investment for plant breeding companies is substantial in both technology and human resources with regards to the costs of phenotyping, maintaining a large training population, and not least the costs of genotyping entire breeding populations. However, genotyping costs are continually decreasing and genotyping of large plant populations is much more manageable today than it was just a few years ago. In a simulation study in tetraploid potato, Slater et al. (2016) estimated that when using a GS training population of 2000 individuals, cost savings around 570,000 Australian dollars could be made compared to a traditional breeding program with high selection intensity, which includes savings due to the smaller breeding population, smaller phenotyping trials, and reduced need for repeat trials to confirm results. This results in breakeven genotyping costs at 100 Australian dollars per genotype in order for GS to be cost-effective (Slater et al. 2016). Simultaneously, they estimated a genetic gain over 20 years more than 5 times as high as the expected genetic gain using phenotypic selection, and even when using merely 500 individuals, genetic gain was estimated to more than double compared to phenotypic selection. Furthermore, Lorenz (2013) and Lin et al. (2016) have found that GS with optimised breeding designs can enhance genetic gain, while consuming less cost per unit time as compared to traditional breeding.

Further cost reductions could be made by utilising historical phenotype data, as it would reduce the costs of establishing, phenotyping, and maintaining initial training populations significantly. If historical data can be correctly adjusted for annual variation of environmental factors, they represent a substantial resource. Indeed, Asoro et al. (2011) found that the prediction accuracy in oat can be increased for some traits when including historical data due to an increased training population. Historical data could thus be used to initiate a GS breeding programme, allowing breeders to realize the potential and benefits of GS, before incorporating contemporary data and recalibrating the model.

In the traditional breeding programme at LKF Vandel, Denmark (LKF Vandel 2016), selections are made up to 4 years after crossing parents, whereas selections with GS can be made as soon as 1 year after crossing. As argued in the paper of Sallam et al. (2015), this means that if only the prediction accuracy exceeds 0.25, GS should surpass phenotypic selection in gain per unit time. Indeed, in this study, the lowest average prediction correlation obtained was 0.30, indicating that even using this small prototype training set, GS has potential to improve breeding efficiency in tetraploid potato for less cost per unit time.

#### Author contribution statement

ES and KLN designed the research and wrote the manuscript; ES performed the genotyping experiments and analysed the data; SB and TA contributed to GBS data processing; EHRS and HØJ contributed to the phenotyping of the MASPOT population; HGK created mapping population at breeding station, provided plant material and performed field experiments; LJ contributed to GBS data analysis.

## Electronic supplementary material

Below is the link to the electronic supplementary material.

Supplementary material 1 (PDF 794 kb)

Supplementary material 2 (PDF 225 kb)

Supplementary material 3 (PDF 190 kb)

Supplementary material 4 (TXT 3481 kb)

Supplementary material 5 (PDF 1386 kb)

Supplementary material 6 (TXT 7 kb)

Supplementary material 7 (TXT 12 kb)
