# Supplementary material for: Genomic prediction of starch content and chipping quality in tetraploid potato using genotyping-by-sequencing
Source: Theor Appl Genet. 2017 Jul 13;130(10):2091–108. doi: 10.1007/s00122-017-2944-y (PMC5606954; doi:10.1007/s00122-017-2944-y)
Supplement: Supplementary file 1 — Supplementary material 1 (PDF 794 kb) [file 122_2017_2944_MOESM1_ESM.pdf]

## Online Resource 1

**Article title:** Genomic prediction of starch content and chipping quality in tetraploid potato using genotyping-by-sequencing

**Journal:** Theoretical and Applied Genetics

**Authors:** Elsa Sverrisdóttir, Stephen Byrne, Ea Høegh Riis Sundmark, Heidi Øllegaard Johnsen, Hanne Grethe Kirk, Torben Asp, Luc Janss, and Kåre L. Nielsen

**Corresponding author:** Elsa Sverrisdóttir, Aalborg University, Department of Chemistry and Bioscience, Fredrik Bajers Vej 7H, 9220 Aalborg, Email: esv@bio.aau.dk; Telephone number: +45 5055 3092

## List of clones in MASPOT population

The MASPOT project (2012-2017) is funded by The Danish Council for Strategic Research (Research grant # 11-116190). The MASPOT population consists of about 5000 offspring that were generated by systematic cross pollination of 18 distinct potato cultivars, either established varieties or advanced breeding clones. The offspring were grown and harvested in field trials at Vandel. Denmark in 2013 and again in 2014 in duplicates. The following is a list of 755 clones that were randomly chosen from the MASPOT population to use in the genomic selection study, along with names of the parents and phenotypic data for chipping quality and starch content. For each trait, there are two values: the average of the raw phenotypic data, and the average of corrected data. These were the 755 clones that remained after data processing due to missing genotype data.

| Offspring | Mother | Father | Chipping quality [scale 1-9] | | Starch content [%] | |
|---|---|---|---|---|---|---|
| | | | Raw average | Corrected | Raw average | Corrected |
| 12-301-01 | 07-LJE-1 | 05-GQE-02 | 5 | 4.57 | 16.68 | 18.33 |
| 12-302-11 | 07-LJE-1 | 89-BJQ-4 | 7 | 6.57 | 22.15 | 23.83 |
| 12-302-13 | 07-LJE-1 | 89-BJQ-4 | 8 | 7.57 | 13.08 | 14.71 |
| 12-302-31 | 07-LJE-1 | 89-BJQ-4 | 7 | 6.57 | 16.3 | 17.96 |
| 12-302-36 | 07-LJE-1 | 89-BJQ-4 | 7 | 6.57 | 17.86 | 19.53 |
| 12-303-04 | 07-LJE-1 | 93-CAQ-14 | 4 | 3.57 | 15.18 | 16.83 |
| 12-303-10 | 07-LJE-1 | 93-CAQ-14 | 4 | 3.57 | 15.96 | 17.61 |
| 12-303-19 | 07-LJE-1 | 93-CAQ-14 | 6 | 5.57 | 18.44 | 20.13 |
| 12-303-28 | 07-LJE-1 | 93-CAQ-14 | 5 | 4.57 | 15.11 | 16.76 |
| 12-303-38 | 07-LJE-1 | 93-CAQ-14 | 7 | 6.57 | 20.94 | 22.63 |
| 12-303-41 | 07-LJE-1 | 93-CAQ-14 | 6 | 5.57 | 14.68 | 16.33 |
| 12-303-44 | 07-LJE-1 | 93-CAQ-14 | 6 | 5.57 | 19.11 | 20.78 |
| 12-303-53 | 07-LJE-1 | 93-CAQ-14 | 7 | 6.57 | 19.7 | 21.38 |
| 12-303-63 | 07-LJE-1 | 93-CAQ-14 | 8 | 7.57 | 18.19 | 19.86 |
| 12-303-67 | 07-LJE-1 | 93-CAQ-14 | NA | NA | 15.33 | 16.98 |
| 12-303-68 | 07-LJE-1 | 93-CAQ-14 | 8 | 7.57 | 18.76 | 20.43 |
| 12-304-06 | 07-LJE-1 | 96-BYM-8 | 5 | 4.57 | 14.5 | 16.16 |
| 12-304-18 | 07-LJE-1 | 96-BYM-8 | 5 | 4.57 | 14.36 | 16.01 |
| 12-304-20 | 07-LJE-1 | 96-BYM-8 | 6 | 5.57 | 12.35 | 13.98 |
| 12-304-25 | 07-LJE-1 | 96-BYM-8 | 6 | 5.57 | 11.1 | 12.73 |
| 12-304-26 | 07-LJE-1 | 96-BYM-8 | 4 | 3.57 | 14.71 | 16.4 |
| 12-304-29 | 07-LJE-1 | 96-BYM-8 | 7 | 6.57 | 12.19 | 13.83 |
| 12-304-35 | 07-LJE-1 | 96-BYM-8 | 4 | 3.57 | 12.49 | 14.13 |
| 12-304-37 | 07-LJE-1 | 96-BYM-8 | 5 | 4.57 | 12.15 | 13.78 |
| 12-304-42 | 07-LJE-1 | 96-BYM-8 | 5 | 4.57 | 15.53 | 17.18 |
| 12-304-48 | 07-LJE-1 | 96-BYM-8 | 4 | 3.57 | 15.18 | 16.83 |
| 12-304-55 | 07-LJE-1 | 96-BYM-8 | 8 | 7.57 | 20.99 | 22.68 |
| 12-304-68 | 07-LJE-1 | 96-BYM-8 | 7 | 6.57 | 14.93 | 16.58 |
| 12-305-06 | 07-LJE-1 | Florice | NA | NA | 10.21 | 11.9 |
| 12-305-10 | 07-LJE-1 | Florice | 4 | 3.57 | 16.91 | 18.58 |

| | | | | | | |
|---|---|---|---|---|---|---|
| 12-305-15 | 07-LJE-1 | Florice | 5 | 4.57 | 14.36 | 16.01 |
| 12-305-34 | 07-LJE-1 | Florice | 7 | 6.57 | 16.37 | 18.03 |
| 12-306-05 | 07-LJE-1 | Jutlandia | 5 | 4.57 | 16.02 | 17.68 |
| 12-306-10 | 07-LJE-1 | Jutlandia | 4 | 3.57 | 15.01 | 16.66 |
| 12-306-31 | 07-LJE-1 | Jutlandia | 4 | 3.57 | 13.34 | 14.98 |
| 12-306-40 | 07-LJE-1 | Jutlandia | 4 | 3.57 | 12.45 | 14.08 |
| 12-306-43 | 07-LJE-1 | Jutlandia | 3 | 2.57 | 13.76 | 15.41 |
| 12-306-48 | 07-LJE-1 | Jutlandia | 6 | 5.57 | 18.16 | 19.83 |
| 12-306-66 | 07-LJE-1 | Jutlandia | 4 | 3.57 | 16.8 | 18.46 |
| 12-306-67 | 07-LJE-1 | Jutlandia | 4 | 3.57 | 15.56 | 17.21 |
| 12-306-68 | 07-LJE-1 | Jutlandia | 6 | 5.57 | 14.78 | 16.43 |
| 12-307-02 | 07-LJE-1 | Rywal | 7 | 6.57 | 16.05 | 17.71 |
| 12-307-03 | 07-LJE-1 | Rywal | 3 | 2.57 | 17.64 | 19.31 |
| 12-307-05 | 07-LJE-1 | Rywal | 4 | 3.57 | 15.83 | 17.48 |
| 12-307-07 | 07-LJE-1 | Rywal | 3 | 2.57 | 14.63 | 16.28 |
| 12-307-14 | 07-LJE-1 | Rywal | 4 | 3.57 | 17.52 | 19.18 |
| 12-307-15 | 07-LJE-1 | Rywal | 3 | 2.57 | 16.98 | 18.63 |
| 12-307-20 | 07-LJE-1 | Rywal | 7 | 6.57 | 19.08 | 20.76 |
| 12-307-27 | 07-LJE-1 | Rywal | 6 | 5.57 | 17.56 | 19.23 |
| 12-307-30 | 07-LJE-1 | Rywal | 8 | 7.57 | 18.54 | 20.21 |
| 12-307-36 | 07-LJE-1 | Rywal | 6 | 5.57 | 15.67 | 17.33 |
| 12-308-13 | 07-LJE-1 | Sarpo_Mira | 5 | 4.57 | 17.59 | 19.26 |
| 12-308-18 | 07-LJE-1 | Sarpo_Mira | 4 | 3.57 | 16.88 | 18.53 |
| 12-308-20 | 07-LJE-1 | Sarpo_Mira | 6 | 5.57 | 11.44 | 13.08 |
| 12-308-21 | 07-LJE-1 | Sarpo_Mira | 4 | 3.57 | 16.04 | 17.68 |
| 12-308-23 | 07-LJE-1 | Sarpo_Mira | 7 | 6.57 | 18.07 | 19.73 |
| 12-308-40 | 07-LJE-1 | Sarpo_Mira | 5 | 4.57 | 17.33 | 18.98 |
| 12-308-41 | 07-LJE-1 | Sarpo_Mira | 4 | 3.57 | 13.24 | 14.88 |
| 12-308-45 | 07-LJE-1 | Sarpo_Mira | 7 | 6.57 | 18.73 | 20.41 |
| 12-309-16 | 04-GIV-03 | 05-GQE-02 | NA | NA | 18.54 | 20.21 |
| 12-309-17 | 04-GIV-03 | 05-GQE-02 | NA | NA | 14.24 | 15.88 |
| 12-309-19 | 04-GIV-03 | 05-GQE-02 | NA | NA | 14.34 | 15.98 |
| 12-309-20 | 04-GIV-03 | 05-GQE-02 | NA | NA | 15.74 | 17.38 |
| 12-309-30 | 04-GIV-03 | 05-GQE-02 | NA | NA | 19.19 | 20.86 |
| 12-309-76 | 04-GIV-03 | 05-GQE-02 | NA | NA | 18.32 | 19.98 |
| 12-310-05 | 04-GIV-03 | 89-BJQ-4 | NA | NA | 19.82 | 21.48 |
| 12-311-08 | 04-GIV-03 | 93-CAQ-14 | NA | NA | 14.68 | 16.33 |
| 12-311-10 | 04-GIV-03 | 93-CAQ-14 | NA | NA | 16.22 | 17.88 |
| 12-311-19 | 04-GIV-03 | 93-CAQ-14 | NA | NA | 16.23 | 17.88 |
| 12-311-20 | 04-GIV-03 | 93-CAQ-14 | NA | NA | 13.84 | 15.48 |
| 12-311-26 | 04-GIV-03 | 93-CAQ-14 | NA | NA | 15.27 | 16.93 |
| 12-311-28 | 04-GIV-03 | 93-CAQ-14 | NA | NA | 11.27 | 12.91 |
| 12-311-43 | 04-GIV-03 | 93-CAQ-14 | NA | NA | 18.42 | 20.08 |

| | | | | | | |
|---|---|---|---|---|---|---|
| 12-312-08 | 04-GIV-03 | 96-BYM-8 | NA | NA | 16.85 | 18.51 |
| 12-312-16 | 04-GIV-03 | 96-BYM-8 | NA | NA | 15.39 | 17.03 |
| 12-312-28 | 04-GIV-03 | 96-BYM-8 | NA | NA | 16.17 | 17.83 |
| 12-312-29 | 04-GIV-03 | 96-BYM-8 | NA | NA | 15.36 | 17.01 |
| 12-312-39 | 04-GIV-03 | 96-BYM-8 | NA | NA | 14.99 | 16.63 |
| 12-312-41 | 04-GIV-03 | 96-BYM-8 | NA | NA | 12.79 | 14.43 |
| 12-312-53 | 04-GIV-03 | 96-BYM-8 | NA | NA | 14.34 | 15.98 |
| 12-312-55 | 04-GIV-03 | 96-BYM-8 | NA | NA | 15.98 | 17.63 |
| 12-312-60 | 04-GIV-03 | 96-BYM-8 | NA | NA | 14.83 | 16.48 |
| 12-312-65 | 04-GIV-03 | 96-BYM-8 | NA | NA | 6.7 | 8.4 |
| 12-313-08 | 04-GIV-03 | Desiree | NA | NA | 18.32 | 19.98 |
| 12-313-23 | 04-GIV-03 | Desiree | NA | NA | 15.11 | 16.76 |
| 12-313-25 | 04-GIV-03 | Desiree | NA | NA | 18.02 | 19.68 |
| 12-313-27 | 04-GIV-03 | Desiree | NA | NA | 16.38 | 18.03 |
| 12-313-51 | 04-GIV-03 | Desiree | NA | NA | 16.9 | 18.56 |
| 12-313-59 | 04-GIV-03 | Desiree | NA | NA | 16.82 | 18.48 |
| 12-313-75 | 04-GIV-03 | Desiree | 4 | 3.57 | 16.74 | 18.41 |
| 12-313-76 | 04-GIV-03 | Desiree | 3 | 2.57 | 15.48 | 17.13 |
| 12-313-79 | 04-GIV-03 | Desiree | 3 | 2.57 | 15.63 | 17.28 |
| 12-314-21 | 04-GIV-03 | Florice | NA | NA | 16.57 | 18.23 |
| 12-314-54 | 04-GIV-03 | Florice | NA | NA | 13.98 | 15.63 |
| 12-314-65 | 04-GIV-03 | Florice | NA | NA | 15.53 | 17.18 |
| 12-314-67 | 04-GIV-03 | Florice | NA | NA | 13.54 | 15.18 |
| 12-314-70 | 04-GIV-03 | Florice | NA | NA | 15.08 | 16.73 |
| 12-314-76 | 04-GIV-03 | Florice | NA | NA | 15.28 | 16.93 |
| 12-314-84 | 04-GIV-03 | Florice | NA | NA | 15.03 | 16.68 |
| 12-315-09 | 04-GIV-03 | Rywal | 4 | 3.57 | 17.02 | 18.68 |
| 12-315-16 | 04-GIV-03 | Rywal | 3 | 2.57 | 16.7 | 18.36 |
| 12-315-47 | 04-GIV-03 | Rywal | 3 | 2.57 | 15.03 | 16.68 |
| 12-315-60 | 04-GIV-03 | Rywal | 4 | 3.57 | 17.05 | 18.71 |
| 12-315-63 | 04-GIV-03 | Rywal | 6 | 5.57 | 17.49 | 19.16 |
| 12-315-66 | 04-GIV-03 | Rywal | 7 | 6.57 | 16.6 | 18.26 |
| 12-315-74 | 04-GIV-03 | Rywal | 5 | 4.57 | 17.62 | 19.28 |
| 12-315-75 | 04-GIV-03 | Rywal | 3 | 2.57 | 15.7 | 17.36 |
| 12-315-79 | 04-GIV-03 | Rywal | 5 | 4.57 | 14.53 | 16.18 |
| 12-316-07 | 04-GIV-03 | Sarpo_Mira | 5 | 4.57 | 16.52 | 18.18 |
| 12-316-11 | 04-GIV-03 | Sarpo_Mira | 8 | 7.57 | 16.9 | 18.56 |
| 12-316-17 | 04-GIV-03 | Sarpo_Mira | 7 | 6.57 | 20.23 | 21.91 |
| 12-316-29 | 04-GIV-03 | Sarpo_Mira | 6 | 5.57 | 13.62 | 15.26 |
| 12-316-39 | 04-GIV-03 | Sarpo_Mira | 6 | 5.57 | 17.67 | 19.33 |
| 12-316-67 | 04-GIV-03 | Sarpo_Mira | 8 | 7.57 | 17.59 | 19.26 |
| 12-317-09 | 05-GQE-02 | 96-BYM-8 | NA | NA | 10.61 | 12.23 |
| 12-317-14 | 05-GQE-02 | 96-BYM-8 | NA | NA | 11.35 | 12.98 |

| | | | | | | |
|---|---|---|---|---|---|---|
| 12-317-17 | 05-GQE-02 | 96-BYM-8 | 2 | 1.57 | 10.15 | 11.78 |
| 12-317-26 | 05-GQE-02 | 96-BYM-8 | 2 | 1.57 | 8.96 | 10.58 |
| 12-317-28 | 05-GQE-02 | 96-BYM-8 | 2 | 1.57 | 10.19 | 11.81 |
| 12-317-37 | 05-GQE-02 | 96-BYM-8 | 3 | 2.57 | 11.84 | 13.48 |
| 12-319-18 | 07-LIX-5 | 89-BJQ-4 | 6 | 5.57 | 18.04 | 19.71 |
| 12-319-30 | 07-LIX-5 | 89-BJQ-4 | 5 | 4.57 | 16.05 | 17.71 |
| 12-319-36 | 07-LIX-5 | 89-BJQ-4 | 8 | 7.57 | 16.62 | 18.28 |
| 12-319-43 | 07-LIX-5 | 89-BJQ-4 | 5 | 4.57 | 14.56 | 16.21 |
| 12-319-44 | 07-LIX-5 | 89-BJQ-4 | 4 | 3.57 | 16.27 | 17.93 |
| 12-319-48 | 07-LIX-5 | 89-BJQ-4 | 4 | 3.57 | 17.12 | 18.78 |
| 12-319-49 | 07-LIX-5 | 89-BJQ-4 | 4 | 3.57 | 15.75 | 17.41 |
| 12-319-54 | 07-LIX-5 | 89-BJQ-4 | 7 | 6.57 | 19.65 | 21.33 |
| 12-319-58 | 07-LIX-5 | 89-BJQ-4 | 5 | 4.57 | 15.22 | 16.88 |
| 12-320-01 | 07-LIX-5 | 93-CAQ-14 | 6 | 5.57 | 16.59 | 18.26 |
| 12-320-04 | 07-LIX-5 | 93-CAQ-14 | NA | NA | 14.62 | 16.33 |
| 12-320-11 | 07-LIX-5 | 93-CAQ-14 | 3 | 2.57 | 15.8 | 17.46 |
| 12-320-23 | 07-LIX-5 | 93-CAQ-14 | 7 | 6.57 | 15.8 | 17.46 |
| 12-320-24 | 07-LIX-5 | 93-CAQ-14 | 3 | 2.57 | 11.42 | 13.06 |
| 12-320-34 | 07-LIX-5 | 93-CAQ-14 | 3 | 2.57 | 14.65 | 16.31 |
| 12-320-40 | 07-LIX-5 | 93-CAQ-14 | 3 | 2.57 | 14.73 | 16.38 |
| 12-320-45 | 07-LIX-5 | 93-CAQ-14 | 2 | 1.57 | 12.1 | 13.73 |
| 12-320-53 | 07-LIX-5 | 93-CAQ-14 | 4 | 3.57 | 10.46 | 12.08 |
| 12-320-54 | 07-LIX-5 | 93-CAQ-14 | 6 | 5.57 | 17.56 | 19.23 |
| 12-321-05 | 07-LIX-5 | Florice | 4 | 3.57 | 13.74 | 15.38 |
| 12-322-03 | 07-LIX-5 | Rywal | 5 | 4.57 | 15.03 | 16.68 |
| 12-322-06 | 07-LIX-5 | Rywal | 3 | 2.57 | 14.06 | 15.71 |
| 12-322-10 | 07-LIX-5 | Rywal | 4 | 3.57 | 15.23 | 16.88 |
| 12-322-12 | 07-LIX-5 | Rywal | 3 | 2.57 | 9.89 | 11.51 |
| 12-322-13 | 07-LIX-5 | Rywal | 4 | 3.57 | 13.79 | 15.43 |
| 12-322-22 | 07-LIX-5 | Rywal | 4 | 3.57 | 15.08 | 16.73 |
| 12-322-32 | 07-LIX-5 | Rywal | 3 | 2.57 | 14.36 | 16.01 |
| 12-322-39 | 07-LIX-5 | Rywal | 3 | 2.57 | 14.71 | 16.36 |
| 12-322-45 | 07-LIX-5 | Rywal | 6 | 5.57 | 14.13 | 15.78 |
| 12-322-48 | 07-LIX-5 | Rywal | 5 | 4.57 | 14.8 | 16.46 |
| 12-322-52 | 07-LIX-5 | Rywal | 3 | 2.57 | 14.63 | 16.28 |
| 12-322-54 | 07-LIX-5 | Rywal | 4 | 3.57 | 14.51 | 16.16 |
| 12-323-06 | 89-BJQ-4 | 05-GQE-02 | NA | NA | 15.28 | 16.93 |
| 12-323-13 | 89-BJQ-4 | 05-GQE-02 | 6 | 5.57 | 11.87 | 13.51 |
| 12-323-17 | 89-BJQ-4 | 05-GQE-02 | 5 | 4.57 | 17.63 | 19.28 |
| 12-323-18 | 89-BJQ-4 | 05-GQE-02 | 5 | 4.57 | 16.1 | 17.76 |
| 12-323-29 | 89-BJQ-4 | 05-GQE-02 | 8 | 7.57 | 18.41 | 20.08 |
| 12-324-07 | 89-BJQ-4 | 93-CAQ-14 | 7 | 6.57 | 16.53 | 18.21 |
| 12-324-09 | 89-BJQ-4 | 93-CAQ-14 | 4 | 3.57 | 17.37 | 19.03 |

| | | | | | | |
|---|---|---|---|---|---|---|
| 12-324-13 | 89-BJQ-4 | 93-CAQ-14 | 5 | 4.57 | 17.19 | 18.86 |
| 12-324-14 | 89-BJQ-4 | 93-CAQ-14 | 2 | 1.57 | 15.72 | 17.38 |
| 12-325-13 | 89-BJQ-4 | Florice | NA | NA | 15.22 | 16.88 |
| 12-325-16 | 89-BJQ-4 | Florice | NA | NA | 15.7 | 17.36 |
| 12-325-20 | 89-BJQ-4 | Florice | NA | NA | 14.69 | 16.36 |
| 12-325-30 | 89-BJQ-4 | Florice | NA | NA | 13.72 | 15.36 |
| 12-325-39 | 89-BJQ-4 | Florice | NA | NA | 17.09 | 18.76 |
| 12-325-40 | 89-BJQ-4 | Florice | NA | NA | 17.04 | 18.71 |
| 12-326-01 | 89-BJQ-4 | Sarpo_Mira | NA | NA | 17.02 | 18.68 |
| 12-326-07 | 89-BJQ-4 | Sarpo_Mira | NA | NA | 15.54 | 17.18 |
| 12-326-13 | 89-BJQ-4 | Sarpo_Mira | NA | NA | 16.92 | 18.63 |
| 12-326-18 | 89-BJQ-4 | Sarpo_Mira | NA | NA | 17.29 | 18.96 |
| 12-326-19 | 89-BJQ-4 | Sarpo_Mira | NA | NA | 14.25 | 15.88 |
| 12-326-20 | 89-BJQ-4 | Sarpo_Mira | NA | NA | 19.02 | 20.68 |
| 12-327-10 | 93-CAQ-14 | 05-GQE-02 | NA | NA | 14.03 | 15.68 |
| 12-327-13 | 93-CAQ-14 | 05-GQE-02 | NA | NA | 13.04 | 14.68 |
| 12-327-16 | 93-CAQ-14 | 05-GQE-02 | 3 | 2.57 | 12.94 | 14.58 |
| 12-327-20 | 93-CAQ-14 | 05-GQE-02 | 4 | 3.57 | 15.01 | 16.66 |
| 12-328-12 | 93-CAQ-14 | Sarpo_Mira | NA | NA | 18.52 | 24.78 |
| 12-328-19 | 93-CAQ-14 | Sarpo_Mira | NA | NA | 13.11 | 14.76 |
| 12-329-17 | 96-BYM-8 | 04-GIV-03 | NA | NA | 13.23 | 14.88 |
| 12-329-22 | 96-BYM-8 | 04-GIV-03 | NA | NA | 10.4 | 12.03 |
| 12-329-34 | 96-BYM-8 | 04-GIV-03 | 4 | 3.57 | 10.88 | 12.53 |
| 12-329-40 | 96-BYM-8 | 04-GIV-03 | 3 | 2.57 | 11.13 | 12.78 |
| 12-330-09 | 96-BYM-8 | 07-LIX-5 | NA | NA | 8.21 | 9.83 |
| 12-330-22 | 96-BYM-8 | 07-LIX-5 | NA | NA | 11.99 | 13.63 |
| 12-330-41 | 96-BYM-8 | 07-LIX-5 | NA | NA | 9.43 | 11.06 |
| 12-330-43 | 96-BYM-8 | 07-LIX-5 | NA | NA | 7.72 | 9.33 |
| 12-330-52 | 96-BYM-8 | 07-LIX-5 | NA | NA | 5.55 | 7.18 |
| 12-331-06 | 96-BYM-8 | 89-BJQ-4 | 4 | 3.57 | 14.56 | 16.21 |
| 12-331-07 | 96-BYM-8 | 89-BJQ-4 | 3 | 2.57 | 11 | 12.63 |
| 12-331-14 | 96-BYM-8 | 89-BJQ-4 | 4 | 3.57 | 12.48 | 14.13 |
| 12-331-17 | 96-BYM-8 | 89-BJQ-4 | 4 | 3.57 | 9.99 | 11.63 |
| 12-331-20 | 96-BYM-8 | 89-BJQ-4 | 5 | 4.57 | 11.33 | 12.96 |
| 12-332-01 | 96-BYM-8 | 93-CAQ-14 | 5 | 4.57 | 11.44 | 13.08 |
| 12-332-04 | 96-BYM-8 | 93-CAQ-14 | 4 | 3.57 | 14.43 | 16.08 |
| 12-332-16 | 96-BYM-8 | 93-CAQ-14 | 3 | 2.57 | 10.28 | 11.91 |
| 12-332-19 | 96-BYM-8 | 93-CAQ-14 | 3 | 2.57 | 11.63 | 13.26 |
| 12-333-04 | 96-BYM-8 | Aventra | NA | NA | 7.71 | 9.33 |
| 12-333-11 | 96-BYM-8 | Aventra | 3 | 2.57 | 10.37 | 12.01 |
| 12-333-13 | 96-BYM-8 | Aventra | NA | NA | 11.22 | 12.88 |
| 12-333-14 | 96-BYM-8 | Aventra | 5 | 4.57 | 10.54 | 12.18 |
| 12-333-27 | 96-BYM-8 | Aventra | 4 | 3.57 | 5.88 | 14.67 |

| | | | | | | |
|---|---|---|---|---|---|---|
| 12-333-39 | 96-BYM-8 | Aventra | 3 | 2.57 | 10.87 | 12.51 |
| 12-333-41 | 96-BYM-8 | Aventra | NA | NA | 17.56 | 19.28 |
| 12-333-43 | 96-BYM-8 | Aventra | 3 | 2.57 | 9.6 | 11.23 |
| 12-334-07 | 96-BYM-8 | Jutlandia | 3 | 2.57 | 10.19 | 11.83 |
| 12-334-13 | 96-BYM-8 | Jutlandia | 3 | 2.57 | 10 | 11.63 |
| 12-334-20 | 96-BYM-8 | Jutlandia | 4 | 3.57 | 11.94 | 13.58 |
| 12-335-01 | 96-BYM-8 | Rywal | 3 | 2.57 | 9.96 | 11.58 |
| 12-335-02 | 96-BYM-8 | Rywal | 3 | 2.57 | 11.9 | 13.53 |
| 12-335-04 | 96-BYM-8 | Rywal | 3 | 2.57 | 10.34 | 11.96 |
| 12-335-09 | 96-BYM-8 | Rywal | NA | NA | 11.2 | 12.83 |
| 12-335-12 | 96-BYM-8 | Rywal | NA | NA | 12.15 | 13.78 |
| 12-335-14 | 96-BYM-8 | Rywal | NA | NA | 12.89 | 14.53 |
| 12-335-20 | 96-BYM-8 | Rywal | 4 | 3.57 | 10.43 | 12.06 |
| 12-336-10 | Agria | 04-GIV-03 | 3 | 2.57 | 14.3 | 15.93 |
| 12-336-18 | Agria | 04-GIV-03 | NA | NA | 12.65 | 14.28 |
| 12-336-30 | Agria | 04-GIV-03 | 5 | 4.57 | 14.74 | 16.38 |
| 12-336-43 | Agria | 04-GIV-03 | 2 | 1.57 | 16.43 | 18.08 |
| 12-336-58 | Agria | 04-GIV-03 | 7 | 6.57 | 14.29 | 15.93 |
| 12-336-68 | Agria | 04-GIV-03 | 4 | 3.57 | 12.98 | 14.61 |
| 12-336-70 | Agria | 04-GIV-03 | 3 | 2.57 | 15.32 | 16.96 |
| 12-336-72 | Agria | 04-GIV-03 | 4 | 3.57 | 12.05 | 13.68 |
| 12-336-74 | Agria | 04-GIV-03 | 4 | 3.57 | 15.83 | 17.48 |
| 12-336-75 | Agria | 04-GIV-03 | 3 | 2.57 | 14.43 | 16.08 |
| 12-336-76 | Agria | 04-GIV-03 | 5 | 4.57 | 17.27 | 18.93 |
| 12-336-77 | Agria | 04-GIV-03 | 4 | 3.57 | 17.39 | 19.06 |
| 12-338-10 | Agria | 07-LIX-5 | 3 | 2.57 | 14.86 | 16.51 |
| 12-338-15 | Agria | 07-LIX-5 | 3 | 2.57 | 13.83 | 15.48 |
| 12-338-20 | Agria | 07-LIX-5 | 5 | 4.57 | 14.83 | 16.48 |
| 12-338-23 | Agria | 07-LIX-5 | 4 | 3.57 | 15.8 | 17.46 |
| 12-338-26 | Agria | 07-LIX-5 | NA | NA | 13.63 | 15.28 |
| 12-338-34 | Agria | 07-LIX-5 | NA | NA | 14.79 | 16.43 |
| 12-338-35 | Agria | 07-LIX-5 | NA | NA | 13.69 | 15.33 |
| 12-339-01 | Agria | 89-BJQ-4 | NA | NA | 17.42 | 19.08 |
| 12-339-12 | Agria | 89-BJQ-4 | NA | NA | 13.24 | 14.88 |
| 12-339-17 | Agria | 89-BJQ-4 | NA | NA | 15.1 | 16.76 |
| 12-339-35 | Agria | 89-BJQ-4 | 5 | 4.57 | 11.98 | 13.61 |
| 12-339-40 | Agria | 89-BJQ-4 | 3 | 2.57 | 15.77 | 17.43 |
| 12-339-45 | Agria | 89-BJQ-4 | 4 | 3.57 | 15.23 | 16.88 |
| 12-340-04 | Agria | 93-CAQ-14 | 2 | 1.57 | 8.64 | 10.26 |
| 12-340-05 | Agria | 93-CAQ-14 | 2 | 1.57 | 9.03 | 10.66 |
| 12-340-12 | Agria | 93-CAQ-14 | 2 | 1.57 | 10.82 | 12.43 |
| 12-340-13 | Agria | 93-CAQ-14 | 4 | 3.57 | 13.29 | 14.93 |
| 12-340-15 | Agria | 93-CAQ-14 | 4 | 3.57 | 10.42 | 12.06 |

| | | | | | | |
|---|---|---|---|---|---|---|
| 12-341-04 | Agria | 96-BYM-8 | 4 | 3.57 | 12.74 | 14.38 |
| 12-341-05 | Agria | 96-BYM-8 | 2 | 1.57 | 12.37 | 14.01 |
| 12-341-09 | Agria | 96-BYM-8 | 3 | 2.57 | 11.64 | 13.28 |
| 12-341-15 | Agria | 96-BYM-8 | 3 | 2.57 | 10.51 | 12.13 |
| 12-341-18 | Agria | 96-BYM-8 | 4 | 3.57 | 10.48 | 12.11 |
| 12-341-19 | Agria | 96-BYM-8 | 3 | 2.57 | 11.99 | 13.63 |
| 12-341-20 | Agria | 96-BYM-8 | NA | NA | 11.19 | 12.83 |
| 12-341-24 | Agria | 96-BYM-8 | NA | NA | 11.85 | 13.48 |
| 12-342-03 | Agria | Aventra | NA | NA | 14.27 | 15.93 |
| 12-342-16 | Agria | Aventra | NA | NA | 13.67 | 15.33 |
| 12-342-25 | Agria | Aventra | NA | NA | 14.89 | 16.53 |
| 12-343-11 | Agria | Desiree | NA | NA | 13.57 | 15.21 |
| 12-343-20 | Agria | Desiree | NA | NA | 11.62 | 13.26 |
| 12-343-21 | Agria | Desiree | NA | NA | 14.33 | 15.98 |
| 12-344-13 | Agria | Florice | NA | NA | 13.62 | 15.26 |
| 12-344-17 | Agria | Florice | NA | NA | 14.02 | 15.66 |
| 12-344-19 | Agria | Florice | NA | NA | 11.8 | 13.43 |
| 12-344-23 | Agria | Florice | NA | NA | 11.99 | 13.63 |
| 12-344-28 | Agria | Florice | NA | NA | 8.96 | 10.58 |
| 12-344-29 | Agria | Florice | NA | NA | 8.78 | 10.38 |
| 12-344-32 | Agria | Florice | NA | NA | 10.31 | 11.93 |
| 12-344-35 | Agria | Florice | NA | NA | 11.22 | 12.86 |
| 12-344-41 | Agria | Florice | NA | NA | 13.81 | 15.46 |
| 12-345-01 | Agria | Jutlandia | NA | NA | 12.9 | 14.53 |
| 12-345-09 | Agria | Jutlandia | NA | NA | 12.17 | 13.81 |
| 12-345-13 | Agria | Jutlandia | NA | NA | 14.1 | 15.76 |
| 12-345-18 | Agria | Jutlandia | NA | NA | 11.24 | 12.86 |
| 12-345-24 | Agria | Jutlandia | NA | NA | 14.14 | 15.78 |
| 12-345-30 | Agria | Jutlandia | 5 | 4.57 | 12.62 | 14.26 |
| 12-345-34 | Agria | Jutlandia | 5 | 4.57 | 15.86 | 17.51 |
| 12-345-43 | Agria | Jutlandia | 3 | 2.57 | 13.98 | 15.63 |
| 12-345-45 | Agria | Jutlandia | NA | NA | 12.84 | 14.48 |
| 12-346-16 | Agria | Rywal | 6 | 5.57 | 12.14 | 13.78 |
| 12-346-18 | Agria | Rywal | 4 | 3.57 | 13.81 | 15.46 |
| 12-346-32 | Agria | Rywal | 3 | 2.57 | 11.65 | 13.28 |
| 12-346-37 | Agria | Rywal | 6 | 5.57 | 14.18 | 15.83 |
| 12-347-04 | Agria | Sarpo_Mira | 6 | 5.57 | 14.75 | 16.41 |
| 12-347-15 | Agria | Sarpo_Mira | 5 | 4.57 | 14.32 | 15.96 |
| 12-347-29 | Agria | Sarpo_Mira | 6 | 5.57 | 15.77 | 17.43 |
| 12-347-32 | Agria | Sarpo_Mira | 6 | 5.57 | 11.08 | 12.71 |
| 12-347-37 | Agria | Sarpo_Mira | 4 | 3.57 | 15.06 | 16.71 |
| 12-347-40 | Agria | Sarpo_Mira | 7 | 6.57 | 12.91 | 14.56 |
| 12-348-11 | Aventra | 04-GIV-03 | 3 | 2.57 | 19 | 20.68 |

| | | | | | | |
|---|---|---|---|---|---|---|
| 12-348-18 | Aventra | 04-GIV-03 | NA | NA | 19.09 | 20.76 |
| 12-348-30 | Aventra | 04-GIV-03 | NA | NA | 15.06 | 16.71 |
| 12-348-49 | Aventra | 04-GIV-03 | NA | NA | 22.12 | 23.81 |
| 12-349-05 | Aventra | 05-GQE-02 | 4 | 3.57 | 18.37 | 20.03 |
| 12-349-15 | Aventra | 05-GQE-02 | 5 | 4.57 | 17.21 | 18.88 |
| 12-349-22 | Aventra | 05-GQE-02 | 3 | 2.57 | 15.18 | 16.83 |
| 12-349-24 | Aventra | 05-GQE-02 | 3 | 2.57 | 16.33 | 17.98 |
| 12-349-40 | Aventra | 05-GQE-02 | 4 | 3.57 | 13.92 | 15.56 |
| 12-350-01 | Aventra | 89-BJQ-4 | NA | NA | 20 | 21.68 |
| 12-350-02 | Aventra | 89-BJQ-4 | NA | NA | 17.72 | 19.38 |
| 12-350-07 | Aventra | 89-BJQ-4 | NA | NA | 17.93 | 19.58 |
| 12-350-12 | Aventra | 89-BJQ-4 | NA | NA | 14.16 | 15.81 |
| 12-350-26 | Aventra | 89-BJQ-4 | NA | NA | 17.37 | 19.03 |
| 12-350-34 | Aventra | 89-BJQ-4 | NA | NA | 17.03 | 18.68 |
| 12-350-38 | Aventra | 89-BJQ-4 | NA | NA | 19.53 | 21.21 |
| 12-351-02 | Aventra | 93-CAQ-14 | NA | NA | 14.79 | 16.43 |
| 12-351-04 | Aventra | 93-CAQ-14 | NA | NA | 13.64 | 15.28 |
| 12-351-09 | Aventra | 93-CAQ-14 | NA | NA | 16.96 | 18.63 |
| 12-351-14 | Aventra | 93-CAQ-14 | NA | NA | 15.45 | 17.11 |
| 12-351-16 | Aventra | 93-CAQ-14 | NA | NA | 17.22 | 18.88 |
| 12-351-31 | Aventra | 93-CAQ-14 | 4 | 3.57 | 15.9 | 17.56 |
| 12-351-33 | Aventra | 93-CAQ-14 | 3 | 2.57 | 14.14 | 15.78 |
| 12-352-01 | Aventra | Desiree | 3 | 2.57 | 13.72 | 15.36 |
| 12-352-06 | Aventra | Desiree | 5 | 4.57 | 16.37 | 18.03 |
| 12-352-17 | Aventra | Desiree | 3 | 2.57 | 13.89 | 15.53 |
| 12-352-19 | Aventra | Desiree | 3 | 2.57 | 14.14 | 15.78 |
| 12-352-29 | Aventra | Desiree | 4 | 3.57 | 16.57 | 18.23 |
| 12-352-43 | Aventra | Desiree | 3 | 2.57 | 13.39 | 15.03 |
| 12-352-44 | Aventra | Desiree | 4 | 3.57 | 15.78 | 17.43 |
| 12-353-16 | Aventra | Florice | 4 | 3.57 | 10.31 | 11.93 |
| 12-353-24 | Aventra | Florice | 1 | 0.57 | 15.15 | 16.81 |
| 12-354-05 | Aventra | Rywal | 3 | 2.57 | 17.93 | 19.61 |
| 12-354-08 | Aventra | Rywal | 5 | 4.57 | 17.19 | 18.86 |
| 12-354-09 | Aventra | Rywal | 5 | 4.57 | 15.34 | 17.01 |
| 12-354-17 | Aventra | Rywal | NA | NA | 13.99 | 15.63 |
| 12-354-27 | Aventra | Rywal | NA | NA | 16.97 | 18.63 |
| 12-354-38 | Aventra | Rywal | NA | NA | 17.64 | 19.31 |
| 12-354-41 | Aventra | Rywal | NA | NA | 16.45 | 18.11 |
| 12-354-42 | Aventra | Rywal | NA | NA | 15.75 | 17.41 |
| 12-355-19 | Aventra | Sarpo_Mira | NA | NA | 15.53 | 17.18 |
| 12-355-27 | Aventra | Sarpo_Mira | NA | NA | 17.89 | 19.56 |
| 12-355-37 | Aventra | Sarpo_Mira | NA | NA | 16.82 | 18.48 |
| 12-355-38 | Aventra | Sarpo_Mira | NA | NA | 17.69 | 19.36 |

| | | | | | | |
|---|---|---|---|---|---|---|
| 12-355-45 | Aventra | Sarpo_Mira | NA | NA | 16.87 | 18.53 |
| 12-356-13 | Desiree | 05-GQE-02 | NA | NA | 15.25 | 16.91 |
| 12-356-14 | Desiree | 05-GQE-02 | NA | NA | 11.25 | 12.88 |
| 12-356-19 | Desiree | 05-GQE-02 | NA | NA | 12.3 | 13.93 |
| 12-356-22 | Desiree | 05-GQE-02 | NA | NA | 12.77 | 14.41 |
| 12-356-34 | Desiree | 05-GQE-02 | NA | NA | 15.61 | 17.26 |
| 12-356-38 | Desiree | 05-GQE-02 | NA | NA | 15.47 | 17.13 |
| 12-357-15 | Desiree | 89-BJQ-4 | 4 | 3.57 | 18.38 | 20.06 |
| 12-357-19 | Desiree | 89-BJQ-4 | 4 | 3.57 | 16.55 | 18.21 |
| 12-357-23 | Desiree | 89-BJQ-4 | 5 | 4.57 | 13.38 | 15.03 |
| 12-357-35 | Desiree | 89-BJQ-4 | 4 | 3.57 | 14.16 | 15.81 |
| 12-357-36 | Desiree | 89-BJQ-4 | 4 | 3.57 | 13.82 | 15.48 |
| 12-357-42 | Desiree | 89-BJQ-4 | 5 | 4.57 | 16.49 | 18.16 |
| 12-358-07 | Desiree | 93-CAQ-14 | NA | NA | 15.12 | 16.78 |
| 12-358-09 | Desiree | 93-CAQ-14 | 3 | 2.57 | 11.95 | 13.58 |
| 12-358-10 | Desiree | 93-CAQ-14 | 3 | 2.57 | 8.75 | 10.38 |
| 12-358-14 | Desiree | 93-CAQ-14 | 3 | 2.57 | 11.8 | 13.43 |
| 12-358-22 | Desiree | 93-CAQ-14 | NA | NA | 13.42 | 15.06 |
| 12-358-25 | Desiree | 93-CAQ-14 | NA | NA | 14.83 | 16.48 |
| 12-359-07 | Desiree | 96-BYM-8 | NA | NA | 16.15 | 17.81 |
| 12-359-12 | Desiree | 96-BYM-8 | NA | NA | 11.78 | 13.41 |
| 12-359-16 | Desiree | 96-BYM-8 | NA | NA | 10.81 | 12.43 |
| 12-359-30 | Desiree | 96-BYM-8 | NA | NA | 10.98 | 12.61 |
| 12-360-02 | Desiree | Florice | NA | NA | 12.19 | 13.83 |
| 12-360-04 | Desiree | Florice | NA | NA | 12.81 | 14.48 |
| 12-360-11 | Desiree | Florice | NA | NA | 11.47 | 13.11 |
| 12-360-16 | Desiree | Florice | NA | NA | 11.67 | 13.31 |
| 12-360-19 | Desiree | Florice | NA | NA | 10.91 | 12.53 |
| 12-361-07 | Desiree | Jutlandia | 4 | 3.57 | 11.97 | 13.61 |
| 12-361-13 | Desiree | Jutlandia | NA | NA | 13.99 | 15.63 |
| 12-361-36 | Desiree | Jutlandia | NA | NA | 14.69 | 16.33 |
| 12-362-02 | Desiree | Rywal | NA | NA | 11.94 | 13.58 |
| 12-362-03 | Desiree | Rywal | NA | NA | 14.93 | 16.58 |
| 12-362-27 | Desiree | Rywal | NA | NA | 13.47 | 15.11 |
| 12-362-31 | Desiree | Rywal | NA | NA | 12.89 | 14.53 |
| 12-362-32 | Desiree | Rywal | NA | NA | 15.03 | 16.68 |
| 12-362-45 | Desiree | Rywal | NA | NA | 14.88 | 16.53 |
| 12-363-10 | Desiree | Sarpo_Mira | NA | NA | 14.53 | 16.18 |
| 12-363-17 | Desiree | Sarpo_Mira | NA | NA | 15.18 | 16.83 |
| 12-363-30 | Desiree | Sarpo_Mira | NA | NA | 16.21 | 17.88 |
| 12-365-04 | Florice | 93-CAQ-14 | NA | NA | 13.23 | 14.88 |
| 12-365-05 | Florice | 93-CAQ-14 | NA | NA | 15.62 | 17.28 |
| 12-365-11 | Florice | 93-CAQ-14 | NA | NA | 12.92 | 14.56 |

| | | | | | | |
|---|---|---|---|---|---|---|
| 12-365-33 | Florice | 93-CAQ-14 | NA | NA | 9.81 | 11.43 |
| 12-365-34 | Florice | 93-CAQ-14 | NA | NA | 11.72 | 13.36 |
| 12-365-36 | Florice | 93-CAQ-14 | NA | NA | 14.51 | 16.16 |
| 12-365-38 | Florice | 93-CAQ-14 | NA | NA | 11.03 | 12.68 |
| 12-365-45 | Florice | 93-CAQ-14 | 3 | 2.57 | 10.05 | 11.68 |
| 12-366-03 | Florice | 96-BYM-8 | 4 | 3.57 | 10.3 | 11.93 |
| 12-366-05 | Florice | 96-BYM-8 | 3 | 2.57 | 11.53 | 13.18 |
| 12-366-07 | Florice | 96-BYM-8 | 5 | 4.57 | 19.49 | 21.18 |
| 12-366-10 | Florice | 96-BYM-8 | 5 | 4.57 | 11.24 | 12.88 |
| 12-366-12 | Florice | 96-BYM-8 | 2 | 1.57 | 8.09 | 9.71 |
| 12-366-19 | Florice | 96-BYM-8 | 4 | 3.57 | 12.3 | 13.93 |
| 12-366-27 | Florice | 96-BYM-8 | 4 | 3.57 | 8.89 | 10.51 |
| 12-366-31 | Florice | 96-BYM-8 | 3 | 2.57 | 11.1 | 12.73 |
| 12-367-04 | Florice | Rywal | 3 | 2.57 | 12.25 | 13.88 |
| 12-367-05 | Florice | Rywal | 2 | 1.57 | 14.36 | 16.01 |
| 12-367-06 | Florice | Rywal | 3 | 2.57 | 11.22 | 12.86 |
| 12-367-08 | Florice | Rywal | 4 | 3.57 | 14.92 | 16.58 |
| 12-368-03 | Jutlandia | 04-GIV-03 | 4 | 3.57 | 18.09 | 19.76 |
| 12-368-07 | Jutlandia | 04-GIV-03 | 4 | 3.57 | 17.64 | 19.31 |
| 12-368-27 | Jutlandia | 04-GIV-03 | 6 | 5.57 | 15.65 | 17.31 |
| 12-368-29 | Jutlandia | 04-GIV-03 | 6 | 5.57 | 16.4 | 18.06 |
| 12-369-10 | Jutlandia | 05-GQE-02 | 2 | 1.57 | 10.71 | 12.33 |
| 12-369-17 | Jutlandia | 05-GQE-02 | 2 | 1.57 | 11.03 | 12.66 |
| 12-369-24 | Jutlandia | 05-GQE-02 | 3 | 2.57 | 13.36 | 15.01 |
| 12-369-25 | Jutlandia | 05-GQE-02 | 4 | 3.57 | 12.02 | 13.66 |
| 12-369-26 | Jutlandia | 05-GQE-02 | 3 | 2.57 | 12.94 | 14.58 |
| 12-369-39 | Jutlandia | 05-GQE-02 | 4 | 3.57 | 12.78 | 14.43 |
| 12-370-03 | Jutlandia | 89-BJQ-4 | 4 | 3.57 | 15.33 | 16.98 |
| 12-370-06 | Jutlandia | 89-BJQ-4 | 8 | 7.57 | 14.34 | 15.98 |
| 12-370-17 | Jutlandia | 89-BJQ-4 | 7 | 6.57 | 16.64 | 18.31 |
| 12-370-26 | Jutlandia | 89-BJQ-4 | 7 | 6.57 | 17.52 | 19.18 |
| 12-370-27 | Jutlandia | 89-BJQ-4 | 8 | 7.57 | 15.45 | 17.11 |
| 12-370-38 | Jutlandia | 89-BJQ-4 | 4 | 3.57 | 13.88 | 15.53 |
| 12-371-04 | Jutlandia | 93-CAQ-14 | 3 | 2.57 | 13.21 | 14.86 |
| 12-371-05 | Jutlandia | 93-CAQ-14 | 2 | 1.57 | 11.94 | 13.58 |
| 12-371-09 | Jutlandia | 93-CAQ-14 | 2 | 1.57 | 11.35 | 12.98 |
| 12-371-10 | Jutlandia | 93-CAQ-14 | 3 | 2.57 | 13.31 | 14.96 |
| 12-371-11 | Jutlandia | 93-CAQ-14 | 3 | 2.57 | 12.2 | 13.83 |
| 12-371-12 | Jutlandia | 93-CAQ-14 | 4 | 3.57 | 12.37 | 14.01 |
| 12-371-17 | Jutlandia | 93-CAQ-14 | 4 | 3.57 | 16.54 | 18.21 |
| 12-371-20 | Jutlandia | 93-CAQ-14 | 4 | 3.57 | 14.28 | 15.93 |
| 12-371-24 | Jutlandia | 93-CAQ-14 | 3 | 2.57 | 13.11 | 14.76 |
| 12-371-30 | Jutlandia | 93-CAQ-14 | 3 | 2.57 | 13.68 | 15.33 |

| | | | | | | |
|---|---|---|---|---|---|---|
| 12-372-08 | Jutlandia | Aventra | 5 | 4.57 | 17.37 | 19.03 |
| 12-372-14 | Jutlandia | Aventra | 3 | 2.57 | 16.19 | 17.86 |
| 12-372-18 | Jutlandia | Aventra | 4 | 3.57 | 14.13 | 15.78 |
| 12-372-29 | Jutlandia | Aventra | 5 | 4.57 | 16.57 | 18.23 |
| 12-372-33 | Jutlandia | Aventra | 2 | 1.57 | 13.74 | 15.38 |
| 12-372-35 | Jutlandia | Aventra | 2 | 1.57 | 13.96 | 15.61 |
| 12-372-43 | Jutlandia | Aventra | 5 | 4.57 | 16.72 | 18.38 |
| 12-373-11 | Jutlandia | Florice | 3 | 2.57 | 12.27 | 13.91 |
| 12-373-18 | Jutlandia | Florice | 4 | 3.57 | 13.79 | 15.48 |
| 12-373-22 | Jutlandia | Florice | 3 | 2.57 | 9.2 | 10.83 |
| 12-373-24 | Jutlandia | Florice | 4 | 3.57 | 12.07 | 13.71 |
| 12-373-28 | Jutlandia | Florice | NA | NA | 15.65 | 17.33 |
| 12-373-33 | Jutlandia | Florice | 4 | 3.57 | 13.53 | 15.18 |
| 12-373-35 | Jutlandia | Florice | 3 | 2.57 | 11.54 | 13.18 |
| 12-373-43 | Jutlandia | Florice | 3 | 2.57 | 13.38 | 15.03 |
| 12-375-42 | Kuras | 04-GIV-03 | 5 | 4.57 | 19.73 | 21.41 |
| 12-375-77 | Kuras | 04-GIV-03 | 4 | 3.57 | 17.55 | 19.21 |
| 12-376-06 | Kuras | 05-GQE-02 | 3 | 2.57 | 17.39 | 19.06 |
| 12-376-16 | Kuras | 05-GQE-02 | 4 | 3.57 | 17.27 | 18.93 |
| 12-376-20 | Kuras | 05-GQE-02 | 4 | 3.57 | 15.14 | 16.78 |
| 12-376-21 | Kuras | 05-GQE-02 | 3 | 2.57 | 14.01 | 15.66 |
| 12-376-39 | Kuras | 05-GQE-02 | 4 | 3.57 | 14.69 | 16.33 |
| 12-376-40 | Kuras | 05-GQE-02 | 6 | 5.57 | 16.13 | 17.78 |
| 12-376-45 | Kuras | 05-GQE-02 | 4 | 3.57 | 14.19 | 15.83 |
| 12-377-09 | Kuras | 89-BJQ-4 | 6 | 5.57 | 16.69 | 18.36 |
| 12-377-17 | Kuras | 89-BJQ-4 | 4 | 3.57 | 15.77 | 17.43 |
| 12-377-19 | Kuras | 89-BJQ-4 | 8 | 7.57 | 19.95 | 21.63 |
| 12-377-22 | Kuras | 89-BJQ-4 | 4 | 3.57 | 16.89 | 18.56 |
| 12-377-25 | Kuras | 89-BJQ-4 | 7 | 6.57 | 18.21 | 19.88 |
| 12-377-27 | Kuras | 89-BJQ-4 | 7 | 6.57 | 19.41 | 21.08 |
| 12-377-34 | Kuras | 89-BJQ-4 | 6 | 5.57 | 18.69 | 20.36 |
| 12-377-36 | Kuras | 89-BJQ-4 | 6 | 5.57 | 17.29 | 18.96 |
| 12-377-40 | Kuras | 89-BJQ-4 | 4 | 3.57 | 18.83 | 20.51 |
| 12-377-44 | Kuras | 89-BJQ-4 | 5 | 4.57 | 20.88 | 22.56 |
| 12-378-01 | Kuras | 93-CAQ-14 | 5 | 4.57 | 16.3 | 17.96 |
| 12-378-06 | Kuras | 93-CAQ-14 | 3 | 2.57 | 13.31 | 14.96 |
| 12-378-12 | Kuras | 93-CAQ-14 | 4 | 3.57 | 14.75 | 16.41 |
| 12-378-18 | Kuras | 93-CAQ-14 | 3 | 2.57 | 14.55 | 16.21 |
| 12-378-26 | Kuras | 93-CAQ-14 | 2 | 1.57 | 16.32 | 17.98 |
| 12-378-35 | Kuras | 93-CAQ-14 | 4 | 3.57 | 16.42 | 18.08 |
| 12-378-37 | Kuras | 93-CAQ-14 | 5 | 4.57 | 15.78 | 17.43 |
| 12-378-38 | Kuras | 93-CAQ-14 | 4 | 3.57 | 17.15 | 18.81 |
| 12-378-39 | Kuras | 93-CAQ-14 | 3 | 2.57 | 11.85 | 13.48 |

| | | | | | | |
|---|---|---|---|---|---|---|
| 12-378-45 | Kuras | 93-CAQ-14 | 5 | 4.57 | 15.38 | 17.03 |
| 12-379-04 | Kuras | 96-BYM-8 | 4 | 3.57 | 14.04 | 15.68 |
| 12-379-05 | Kuras | 96-BYM-8 | 3 | 2.57 | 12.86 | 14.51 |
| 12-379-06 | Kuras | 96-BYM-8 | 2 | 1.57 | 12.96 | 14.61 |
| 12-379-09 | Kuras | 96-BYM-8 | 4 | 3.57 | 13.44 | 15.08 |
| 12-379-26 | Kuras | 96-BYM-8 | 3 | 2.57 | 13.23 | 14.88 |
| 12-379-27 | Kuras | 96-BYM-8 | 4 | 3.57 | 14.56 | 16.21 |
| 12-379-28 | Kuras | 96-BYM-8 | 4 | 3.57 | 14.36 | 16.01 |
| 12-379-39 | Kuras | 96-BYM-8 | 3 | 2.57 | 13.21 | 14.86 |
| 12-379-40 | Kuras | 96-BYM-8 | 4 | 3.57 | 12.16 | 13.78 |
| 12-380-01 | Kuras | Aventra | 3 | 2.57 | 18.19 | 19.86 |
| 12-380-10 | Kuras | Aventra | 4 | 3.57 | 18.76 | 20.43 |
| 12-380-11 | Kuras | Aventra | 4 | 3.57 | 17.76 | 19.43 |
| 12-380-29 | Kuras | Aventra | 5 | 4.57 | 15.77 | 17.43 |
| 12-380-41 | Kuras | Aventra | 6 | 5.57 | 19.01 | 20.68 |
| 12-381-05 | Kuras | Desiree | 4 | 3.57 | 14.65 | 16.31 |
| 12-381-06 | Kuras | Desiree | 3 | 2.57 | 16.33 | 17.98 |
| 12-381-07 | Kuras | Desiree | 5 | 4.57 | 15.93 | 17.58 |
| 12-381-18 | Kuras | Desiree | 4 | 3.57 | 14.59 | 16.23 |
| 12-381-23 | Kuras | Desiree | 3 | 2.57 | 15.73 | 17.38 |
| 12-381-25 | Kuras | Desiree | 4 | 3.57 | 17.74 | 19.41 |
| 12-381-26 | Kuras | Desiree | 3 | 2.57 | 13.88 | 15.53 |
| 12-381-29 | Kuras | Desiree | 4 | 3.57 | 13.86 | 15.51 |
| 12-381-42 | Kuras | Desiree | 4 | 3.57 | 14.76 | 16.41 |
| 12-382-01 | Kuras | Florice | 3 | 2.57 | 15.68 | 17.33 |
| 12-382-07 | Kuras | Florice | 5 | 4.57 | 13.04 | 14.68 |
| 12-382-08 | Kuras | Florice | 3 | 2.57 | 14.78 | 16.43 |
| 12-382-16 | Kuras | Florice | 2 | 1.57 | 15.13 | 16.78 |
| 12-382-18 | Kuras | Florice | 3 | 2.57 | 11.95 | 13.58 |
| 12-382-36 | Kuras | Florice | 4 | 3.57 | 13.54 | 15.18 |
| 12-383-01 | Kuras | Jutlandia | 5 | 4.57 | 13.46 | 15.11 |
| 12-383-03 | Kuras | Jutlandia | 4 | 3.57 | 14.98 | 16.63 |
| 12-383-05 | Kuras | Jutlandia | 3 | 2.57 | 14.26 | 15.91 |
| 12-383-15 | Kuras | Jutlandia | 4 | 3.57 | 13.79 | 15.43 |
| 12-383-20 | Kuras | Jutlandia | 6 | 5.57 | 14.51 | 16.16 |
| 12-383-24 | Kuras | Jutlandia | 4 | 3.57 | 14.48 | 16.13 |
| 12-384-09 | Kuras | Rywal | 5 | 4.57 | 16.05 | 17.71 |
| 12-384-10 | Kuras | Rywal | 5 | 4.57 | 16.87 | 18.53 |
| 12-384-13 | Kuras | Rywal | 3 | 2.57 | 15.01 | 16.66 |
| 12-384-27 | Kuras | Rywal | 3 | 2.57 | 14.48 | 16.13 |
| 12-384-42 | Kuras | Rywal | 3 | 2.57 | 13.54 | 15.18 |
| 12-384-44 | Kuras | Rywal | 4 | 3.57 | 16.45 | 18.11 |
| 12-384-45 | Kuras | Rywal | 5 | 4.57 | 16.45 | 18.11 |

| | | | | | | |
|---|---|---|---|---|---|---|
| 12-385-02 | Kuras | Sarpo_Mira | 6 | 5.57 | 16.7 | 18.36 |
| 12-385-04 | Kuras | Sarpo_Mira | 6 | 5.57 | 16.5 | 18.16 |
| 12-385-13 | Kuras | Sarpo_Mira | 5 | 4.57 | 15.39 | 17.03 |
| 12-385-17 | Kuras | Sarpo_Mira | 6 | 5.57 | 14.98 | 16.63 |
| 12-385-20 | Kuras | Sarpo_Mira | 4 | 3.57 | 16.87 | 18.53 |
| 12-385-32 | Kuras | Sarpo_Mira | 8 | 7.57 | 16.72 | 18.38 |
| 12-385-35 | Kuras | Sarpo_Mira | 4 | 3.57 | 16.63 | 18.28 |
| 12-385-37 | Kuras | Sarpo_Mira | 6 | 5.57 | 16.6 | 18.26 |
| 12-385-44 | Kuras | Sarpo_Mira | 4 | 3.57 | 15.58 | 17.23 |
| 12-386-13 | Rywal | 04-GIV-03 | 7 | 6.57 | 13.93 | 15.58 |
| 12-386-63 | Rywal | 04-GIV-03 | 4 | 3.57 | 14.9 | 16.56 |
| 12-386-78 | Rywal | 04-GIV-03 | 3 | 2.57 | 17.67 | 19.33 |
| 12-386-82 | Rywal | 04-GIV-03 | 4 | 3.57 | 13.04 | 14.68 |
| 12-387-12 | Rywal | 05-GQE-02 | 3 | 2.57 | 14.86 | 16.51 |
| 12-387-21 | Rywal | 05-GQE-02 | 5 | 4.57 | 15.28 | 16.93 |
| 12-387-32 | Rywal | 05-GQE-02 | 5 | 4.57 | 13.29 | 14.93 |
| 12-387-39 | Rywal | 05-GQE-02 | 4 | 3.57 | 16.05 | 17.71 |
| 12-387-41 | Rywal | 05-GQE-02 | 4 | 3.57 | 13.22 | 14.86 |
| 12-387-42 | Rywal | 05-GQE-02 | 4 | 3.57 | 19.09 | 20.78 |
| 12-388-05 | Rywal | 89-BJQ-4 | 4 | 3.57 | 14.99 | 16.63 |
| 12-388-13 | Rywal | 89-BJQ-4 | 7 | 6.57 | 19.6 | 21.28 |
| 12-388-14 | Rywal | 89-BJQ-4 | 5 | 4.57 | 16.35 | 18.01 |
| 12-388-19 | Rywal | 89-BJQ-4 | 6 | 5.57 | 16.3 | 17.96 |
| 12-389-13 | Rywal | 93-CAQ-14 | 3 | 2.57 | 16.12 | 17.78 |
| 12-389-15 | Rywal | 93-CAQ-14 | 3 | 2.57 | 14.21 | 15.86 |
| 12-389-23 | Rywal | 93-CAQ-14 | 4 | 3.57 | 17.36 | 19.03 |
| 12-390-07 | Rywal | Jutlandia | 4 | 3.57 | 14.65 | 16.31 |
| 12-390-16 | Rywal | Jutlandia | 3 | 2.57 | 11.67 | 13.31 |
| 12-390-20 | Rywal | Jutlandia | 7 | 6.57 | 15.22 | 16.88 |
| 12-391-08 | Sarpo_Mira | 05-GQE-02 | 6 | 5.57 | 12.99 | 14.63 |
| 12-391-14 | Sarpo_Mira | 05-GQE-02 | 5 | 4.57 | 15.16 | 16.81 |
| 12-391-22 | Sarpo_Mira | 05-GQE-02 | 3 | 2.57 | 12.27 | 13.91 |
| 12-391-33 | Sarpo_Mira | 05-GQE-02 | 3 | 2.57 | 13.09 | 14.73 |
| 12-391-38 | Sarpo_Mira | 05-GQE-02 | 3 | 2.57 | 11.87 | 13.51 |
| 12-391-41 | Sarpo_Mira | 05-GQE-02 | 8 | 7.57 | 11.85 | 13.48 |
| 12-391-42 | Sarpo_Mira | 05-GQE-02 | 3 | 2.57 | 13.44 | 15.08 |
| 12-392-02 | Sarpo_Mira | 96-BYM-8 | 5 | 4.57 | 10.45 | 12.08 |
| 12-392-11 | Sarpo_Mira | 96-BYM-8 | 7 | 6.57 | 12.97 | 14.61 |
| 12-392-22 | Sarpo_Mira | 96-BYM-8 | 5 | 4.57 | 14.81 | 16.46 |
| 12-392-37 | Sarpo_Mira | 96-BYM-8 | 4 | 3.57 | 7.26 | 8.88 |
| 12-393-30 | Sarpo_Mira | Florice | 5 | 4.57 | 14.67 | 16.33 |
| 12-393-35 | Sarpo_Mira | Florice | 2 | 1.57 | 12.87 | 14.51 |
| 12-394-08 | Sarpo_Mira | Jutlandia | 3 | 2.57 | 12.7 | 14.33 |

| | | | | | | |
|---|---|---|---|---|---|---|
| 12-394-10 | Sarpo_Mira | Jutlandia | 4 | 3.57 | 16.22 | 17.88 |
| 12-394-11 | Sarpo_Mira | Jutlandia | 4 | 3.57 | 15.13 | 16.78 |
| 12-394-21 | Sarpo_Mira | Jutlandia | 4 | 3.57 | 16.25 | 17.91 |
| 12-394-22 | Sarpo_Mira | Jutlandia | 4 | 3.57 | 11.85 | 13.48 |
| 12-394-27 | Sarpo_Mira | Jutlandia | NA | NA | 19.71 | 21.43 |
| 12-394-30 | Sarpo_Mira | Jutlandia | 4 | 3.57 | 10.88 | 12.51 |
| 12-394-34 | Sarpo_Mira | Jutlandia | 4 | 3.57 | 13.67 | 15.31 |
| 12-394-40 | Sarpo_Mira | Jutlandia | 5 | 4.57 | 13.63 | 15.28 |
| 12-394-42 | Sarpo_Mira | Jutlandia | 5 | 4.57 | 14.14 | 15.78 |
| 12-395-20 | Sarpo_Mira | Rywal | 3 | 2.57 | 14.09 | 15.73 |
| 12-395-39 | Sarpo_Mira | Rywal | 7 | 6.57 | 15.93 | 17.58 |
| 12-395-44 | Sarpo_Mira | Rywal | 4 | 3.57 | 13.44 | 15.08 |
| 12-396-04 | Shepody | 05-GQE-02 | 5 | 4.57 | 13.42 | 15.06 |
| 12-396-06 | Shepody | 05-GQE-02 | 3 | 2.57 | 16.13 | 17.78 |
| 12-396-23 | Shepody | 05-GQE-02 | 3 | 2.57 | 11.46 | 13.08 |
| 12-396-28 | Shepody | 05-GQE-02 | 4 | 3.57 | 13.54 | 15.18 |
| 12-396-35 | Shepody | 05-GQE-02 | 3 | 2.57 | 13 | 14.63 |
| 12-398-01 | Shepody | 89-BJQ-4 | 4 | 3.57 | 17.72 | 19.38 |
| 12-398-02 | Shepody | 89-BJQ-4 | 3 | 2.57 | 13.79 | 15.43 |
| 12-398-06 | Shepody | 89-BJQ-4 | 3 | 2.57 | 15.16 | 16.81 |
| 12-398-10 | Shepody | 89-BJQ-4 | 4 | 3.57 | 11.76 | 13.38 |
| 12-398-17 | Shepody | 89-BJQ-4 | 4 | 3.57 | 10.33 | 11.93 |
| 12-398-20 | Shepody | 89-BJQ-4 | 4 | 3.57 | 16.22 | 17.88 |
| 12-398-25 | Shepody | 89-BJQ-4 | 6 | 5.57 | 18.13 | 19.81 |
| 12-398-26 | Shepody | 89-BJQ-4 | 4 | 3.57 | 14.68 | 16.33 |
| 12-398-38 | Shepody | 89-BJQ-4 | 4 | 3.57 | 16.03 | 17.68 |
| 12-398-43 | Shepody | 89-BJQ-4 | 3 | 2.57 | 15.83 | 17.48 |
| 12-398-44 | Shepody | 89-BJQ-4 | 5 | 4.57 | 16.13 | 17.78 |
| 12-399-03 | Shepody | 93-CAQ-14 | 3 | 2.57 | 12.26 | 13.91 |
| 12-399-11 | Shepody | 93-CAQ-14 | 4 | 3.57 | 12.84 | 14.48 |
| 12-399-23 | Shepody | 93-CAQ-14 | 7 | 6.57 | 14.51 | 16.16 |
| 12-399-26 | Shepody | 93-CAQ-14 | 4 | 3.57 | 10.68 | 12.31 |
| 12-399-28 | Shepody | 93-CAQ-14 | 4 | 3.57 | 14.02 | 15.68 |
| 12-399-35 | Shepody | 93-CAQ-14 | 4 | 3.57 | 13.88 | 15.53 |
| 12-399-37 | Shepody | 93-CAQ-14 | 5 | 4.57 | 14.73 | 16.38 |
| 12-399-39 | Shepody | 93-CAQ-14 | 5 | 4.57 | 14.91 | 16.56 |
| 12-400-21 | Shepody | 96-BYM-8 | NA | NA | 9.96 | 11.58 |
| 12-400-28 | Shepody | 96-BYM-8 | NA | NA | 13.19 | 14.83 |
| 12-400-31 | Shepody | 96-BYM-8 | NA | NA | 9.01 | 10.63 |
| 12-401-02 | Shepody | Florice | NA | NA | 9.6 | 11.17 |
| 12-401-07 | Shepody | Florice | NA | NA | 11.51 | 13.13 |
| 12-401-14 | Shepody | Florice | NA | NA | 11.5 | 13.13 |
| 12-401-22 | Shepody | Florice | NA | NA | 11.82 | 13.46 |

| | | | | | | |
|---|---|---|---|---|---|---|
| 12-401-37 | Shepody | Florice | NA | NA | 10.48 | 12.11 |
| 12-402-01 | Shepody | Jutlandia | NA | NA | 13.43 | 15.08 |
| 12-402-09 | Shepody | Jutlandia | NA | NA | 13.74 | 15.38 |
| 12-402-35 | Shepody | Jutlandia | NA | NA | 14.41 | 16.06 |
| 12-402-40 | Shepody | Jutlandia | NA | NA | 12.99 | 14.63 |
| 12-404-06 | Shepody | Rywal | 4 | 3.57 | 13.91 | 15.56 |
| 12-404-07 | Shepody | Rywal | 4 | 3.57 | 14.53 | 16.18 |
| 12-404-12 | Shepody | Rywal | 3 | 2.57 | 12.14 | 13.78 |
| 12-404-27 | Shepody | Rywal | 4 | 3.57 | 14.84 | 16.48 |
| 12-404-37 | Shepody | Rywal | 6 | 5.57 | 14.11 | 15.76 |
| 12-404-40 | Shepody | Rywal | 4 | 3.57 | 16.75 | 18.43 |
| 12-406-14 | Shepody | Sarpo_Mira | 4 | 3.57 | 15.46 | 17.11 |
| 12-406-16 | Shepody | Sarpo_Mira | 7 | 6.57 | 21.81 | 23.53 |
| 12-407-02 | Spunta | 05-GQE-02 | 5 | 4.57 | 13.19 | 14.83 |
| 12-407-13 | Spunta | 05-GQE-02 | NA | NA | 16.61 | 18.28 |
| 12-407-14 | Spunta | 05-GQE-02 | 7 | 6.57 | 12.4 | 14.03 |
| 12-407-16 | Spunta | 05-GQE-02 | 5 | 4.57 | 11.58 | 13.21 |
| 12-407-23 | Spunta | 05-GQE-02 | NA | NA | 11.55 | 13.18 |
| 12-407-24 | Spunta | 05-GQE-02 | NA | NA | 13.05 | 14.68 |
| 12-407-25 | Spunta | 05-GQE-02 | 3 | 2.57 | 12.6 | 14.23 |
| 12-407-30 | Spunta | 05-GQE-02 | 2 | 1.57 | 9.51 | 11.13 |
| 12-407-33 | Spunta | 05-GQE-02 | 4 | 3.57 | 11.85 | 13.48 |
| 12-407-42 | Spunta | 05-GQE-02 | 4 | 3.57 | 14.86 | 16.51 |
| 12-408-01 | Spunta | 89-BJQ-4 | 5 | 4.57 | 13.14 | 14.78 |
| 12-408-06 | Spunta | 89-BJQ-4 | 6 | 5.57 | 13.34 | 14.98 |
| 12-408-07 | Spunta | 89-BJQ-4 | 6 | 5.57 | 14.74 | 16.38 |
| 12-408-09 | Spunta | 89-BJQ-4 | 7 | 6.57 | 12.94 | 14.58 |
| 12-408-19 | Spunta | 89-BJQ-4 | 3 | 2.57 | 13.94 | 15.58 |
| 12-408-24 | Spunta | 89-BJQ-4 | 3 | 2.57 | 13.66 | 15.31 |
| 12-408-27 | Spunta | 89-BJQ-4 | 4 | 3.57 | 15.73 | 17.38 |
| 12-408-36 | Spunta | 89-BJQ-4 | 5 | 4.57 | 16.57 | 18.23 |
| 12-408-43 | Spunta | 89-BJQ-4 | 5 | 4.57 | 15.04 | 16.68 |
| 12-409-23 | Spunta | 93-CAQ-14 | 3 | 2.57 | 9.93 | 11.56 |
| 12-409-29 | Spunta | 93-CAQ-14 | 3 | 2.57 | 9.97 | 11.63 |
| 12-409-34 | Spunta | 93-CAQ-14 | 5 | 4.57 | 13.66 | 15.31 |
| 12-410-10 | Spunta | 96-BYM-8 | 4 | 3.57 | 11.05 | 12.68 |
| 12-410-20 | Spunta | 96-BYM-8 | 3 | 2.57 | 10.33 | 11.96 |
| 12-410-27 | Spunta | 96-BYM-8 | 4 | 3.57 | 9.02 | 10.63 |
| 12-410-28 | Spunta | 96-BYM-8 | 4 | 3.57 | 11.02 | 12.66 |
| 12-410-37 | Spunta | 96-BYM-8 | 3 | 2.57 | 10.13 | 11.76 |
| 12-410-38 | Spunta | 96-BYM-8 | 3 | 2.57 | 9.93 | 11.56 |
| 12-411-01 | Spunta | Aventra | 2 | 1.57 | 13.51 | 15.16 |
| 12-411-08 | Spunta | Aventra | 3 | 2.57 | 14.98 | 16.63 |

| | | | | | | |
|---|---|---|---|---|---|---|
| 12-411-14 | Spunta | Aventra | 3 | 2.57 | 15.63 | 17.28 |
| 12-411-18 | Spunta | Aventra | 4 | 3.57 | 17.49 | 19.16 |
| 12-411-32 | Spunta | Aventra | 4 | 3.57 | 16.84 | 18.51 |
| 12-412-13 | Spunta | Desiree | NA | NA | 12.09 | 13.73 |
| 12-412-17 | Spunta | Desiree | NA | NA | 13.17 | 14.81 |
| 12-412-23 | Spunta | Desiree | NA | NA | 13.16 | 14.81 |
| 12-412-42 | Spunta | Desiree | 3 | 2.57 | 11.03 | 12.66 |
| 12-412-45 | Spunta | Desiree | 2 | 1.57 | 10.43 | 12.06 |
| 12-413-06 | Spunta | Florice | 3 | 2.57 | 11.09 | 12.73 |
| 12-413-09 | Spunta | Florice | NA | NA | 13.01 | 14.66 |
| 12-413-10 | Spunta | Florice | NA | NA | 12.69 | 14.33 |
| 12-413-12 | Spunta | Florice | 4 | 3.57 | 12.99 | 14.63 |
| 12-413-13 | Spunta | Florice | NA | NA | 10.18 | 11.81 |
| 12-413-14 | Spunta | Florice | 6 | 5.57 | 12.46 | 14.11 |
| 12-413-16 | Spunta | Florice | 7 | 6.57 | 11.32 | 12.96 |
| 12-413-18 | Spunta | Florice | 2 | 1.57 | 4.91 | 6.53 |
| 12-414-03 | Spunta | Jutlandia | 4 | 3.57 | 10.72 | 12.36 |
| 12-414-09 | Spunta | Jutlandia | 4 | 3.57 | 11.05 | 12.68 |
| 12-414-19 | Spunta | Jutlandia | 3 | 2.57 | 12.25 | 13.88 |
| 12-414-26 | Spunta | Jutlandia | NA | NA | 12.88 | 14.53 |
| 12-414-30 | Spunta | Jutlandia | 4 | 3.57 | 14.19 | 15.83 |
| 12-414-37 | Spunta | Jutlandia | 3 | 2.57 | 10.62 | 12.26 |
| 12-414-38 | Spunta | Jutlandia | 7 | 6.57 | 13.83 | 15.48 |
| 12-415-01 | Spunta | Rywal | 5 | 4.57 | 13.84 | 15.48 |
| 12-415-02 | Spunta | Rywal | 3 | 2.57 | 14.09 | 15.73 |
| 12-415-03 | Spunta | Rywal | 7 | 6.57 | 15.93 | 17.58 |
| 12-415-07 | Spunta | Rywal | 3 | 2.57 | 11.3 | 12.93 |
| 12-415-08 | Spunta | Rywal | 4 | 3.57 | 14.04 | 15.68 |
| 12-415-09 | Spunta | Rywal | NA | NA | 14.91 | 16.6 |
| 12-415-11 | Spunta | Rywal | 3 | 2.57 | 11.54 | 13.18 |
| 12-415-14 | Spunta | Rywal | 4 | 3.57 | 14.92 | 16.58 |
| 12-415-15 | Spunta | Rywal | 5 | 4.57 | 11.62 | 13.26 |
| 12-415-22 | Spunta | Rywal | 4 | 3.57 | 12.45 | 14.08 |
| 12-415-34 | Spunta | Rywal | 5 | 4.57 | 13.19 | 14.83 |
| 12-415-36 | Spunta | Rywal | 3 | 2.57 | 11.68 | 13.31 |
| 12-415-44 | Spunta | Rywal | 4 | 3.57 | 16.02 | 17.68 |
| 12-416-22 | Spunta | Sarpo_Mira | 3 | 2.57 | 11.98 | 13.61 |
| 12-416-23 | Spunta | Sarpo_Mira | 4 | 3.57 | 9.15 | 10.76 |
| 12-416-33 | Spunta | Sarpo_Mira | 3 | 2.57 | 11.23 | 12.86 |
| 12-416-36 | Spunta | Sarpo_Mira | 2 | 1.57 | 10.46 | 12.08 |
| 12-416-44 | Spunta | Sarpo_Mira | 4 | 3.57 | 13.96 | 15.61 |
| 12-417-05 | Spunta | Isle_of_Jura | 4 | 3.57 | 13.51 | 15.16 |
| 12-417-10 | Spunta | Isle_of_Jura | 3 | 2.57 | 10.79 | 12.41 |

| | | | | | | |
|---|---|---|---|---|---|---|
| 12-417-12 | Spunta | Isle_of_Jura | 3 | 2.57 | 14.98 | 16.63 |
| 12-417-16 | Spunta | Isle_of_Jura | 4 | 3.57 | 11 | 12.63 |
| 12-417-17 | Spunta | Isle_of_Jura | 3 | 2.57 | 11.58 | 13.21 |
| 12-417-19 | Spunta | Isle_of_Jura | 3 | 2.57 | 11.93 | 13.56 |
| 12-417-22 | Spunta | Isle_of_Jura | 3 | 2.57 | 10.58 | 12.21 |
| 12-417-23 | Spunta | Isle_of_Jura | 4 | 3.57 | 11.99 | 13.63 |
| 12-417-31 | Spunta | Isle_of_Jura | 3 | 2.57 | 11.75 | 13.38 |
| 12-417-33 | Spunta | Isle_of_Jura | 4 | 3.57 | 11.05 | 12.68 |
| 12-417-39 | Spunta | Isle_of_Jura | 5 | 4.57 | 12.21 | 13.83 |
| 12-418-06 | Isle_of_Jura | 05-GQE-02 | 3 | 2.57 | 12.4 | 14.03 |
| 12-418-19 | Isle_of_Jura | 05-GQE-02 | 4 | 3.57 | 15.15 | 16.81 |
| 12-418-22 | Isle_of_Jura | 05-GQE-02 | 4 | 3.57 | 12.15 | 13.78 |
| 12-418-23 | Isle_of_Jura | 05-GQE-02 | 5 | 4.57 | 10.71 | 12.33 |
| 12-418-39 | Isle_of_Jura | 05-GQE-02 | 4 | 3.57 | 15.31 | 16.96 |
| 12-418-43 | Isle_of_Jura | 05-GQE-02 | 2 | 1.57 | 12.59 | 14.23 |
| 12-418-45 | Isle_of_Jura | 05-GQE-02 | 3 | 2.57 | 9.9 | 11.51 |
| 12-419-04 | Isle_of_Jura | 89-BJQ-4 | NA | NA | 13.38 | 15.03 |
| 12-419-07 | Isle_of_Jura | 89-BJQ-4 | NA | NA | 14.47 | 16.11 |
| 12-419-08 | Isle_of_Jura | 89-BJQ-4 | NA | NA | 18.09 | 19.76 |
| 12-419-19 | Isle_of_Jura | 89-BJQ-4 | NA | NA | 17.05 | 18.71 |
| 12-419-24 | Isle_of_Jura | 89-BJQ-4 | 3 | 2.57 | 13.34 | 14.98 |
| 12-419-31 | Isle_of_Jura | 89-BJQ-4 | NA | NA | 15.75 | 17.41 |
| 12-419-32 | Isle_of_Jura | 89-BJQ-4 | NA | NA | 16.86 | 18.51 |
| 12-419-39 | Isle_of_Jura | 89-BJQ-4 | NA | NA | 18.12 | 19.81 |
| 12-419-40 | Isle_of_Jura | 89-BJQ-4 | NA | NA | 14.66 | 16.31 |
| 12-419-44 | Isle_of_Jura | 89-BJQ-4 | NA | NA | 15.45 | 17.11 |
| 12-420-01 | Isle_of_Jura | 93-CAQ-14 | NA | NA | 10.15 | 11.78 |
| 12-420-02 | Isle_of_Jura | 93-CAQ-14 | NA | NA | 12.94 | 14.58 |
| 12-420-16 | Isle_of_Jura | 93-CAQ-14 | NA | NA | 12.59 | 14.23 |
| 12-420-19 | Isle_of_Jura | 93-CAQ-14 | NA | NA | 10.69 | 12.33 |
| 12-420-32 | Isle_of_Jura | 93-CAQ-14 | 1 | 0.57 | 9.71 | 11.33 |
| 12-420-35 | Isle_of_Jura | 93-CAQ-14 | 5 | 4.57 | 16.06 | 17.73 |
| 12-420-38 | Isle_of_Jura | 93-CAQ-14 | 3 | 2.57 | 14.29 | 15.93 |
| 12-421-09 | Isle_of_Jura | 96-BYM-8 | 4 | 3.57 | 13.74 | 15.38 |
| 12-421-11 | Isle_of_Jura | 96-BYM-8 | 3 | 2.57 | 10.19 | 11.81 |
| 12-421-14 | Isle_of_Jura | 96-BYM-8 | 4 | 3.57 | 9.98 | 11.61 |
| 12-421-20 | Isle_of_Jura | 96-BYM-8 | 3 | 2.57 | 8.26 | 9.88 |
| 12-421-21 | Isle_of_Jura | 96-BYM-8 | 7 | 6.57 | 9.59 | 11.21 |
| 12-421-26 | Isle_of_Jura | 96-BYM-8 | 3 | 2.57 | 10.36 | 11.98 |
| 12-421-41 | Isle_of_Jura | 96-BYM-8 | 4 | 3.57 | 12.04 | 13.68 |
| 12-421-44 | Isle_of_Jura | 96-BYM-8 | 3 | 2.57 | 10.91 | 12.53 |
| 12-423-08 | Isle_of_Jura | Desiree | 4 | 3.57 | 13.99 | 15.63 |
| 12-423-12 | Isle_of_Jura | Desiree | 6 | 5.57 | 13.98 | 15.63 |

| | | | | | | |
|---|---|---|---|---|---|---|
| 12-423-25 | Isle_of_Jura | Desiree | 4 | 3.57 | 15.75 | 17.41 |
| 12-423-32 | Isle_of_Jura | Desiree | 3 | 2.57 | 12.56 | 14.21 |
| 12-423-33 | Isle_of_Jura | Desiree | 3 | 2.57 | 11.56 | 13.18 |
| 12-423-39 | Isle_of_Jura | Desiree | 2 | 1.57 | 9.15 | 10.78 |
| 12-424-07 | Isle_of_Jura | Florice | NA | NA | 11.1 | 12.73 |
| 12-424-11 | Isle_of_Jura | Florice | NA | NA | 12.34 | 13.98 |
| 12-424-14 | Isle_of_Jura | Florice | NA | NA | 14.95 | 16.61 |
| 12-424-18 | Isle_of_Jura | Florice | NA | NA | 13.59 | 15.23 |
| 12-424-21 | Isle_of_Jura | Florice | NA | NA | 13.04 | 14.68 |
| 12-424-23 | Isle_of_Jura | Florice | NA | NA | 11.25 | 12.88 |
| 12-424-25 | Isle_of_Jura | Florice | NA | NA | 11.28 | 12.91 |
| 12-424-28 | Isle_of_Jura | Florice | NA | NA | 13.04 | 14.68 |
| 12-424-33 | Isle_of_Jura | Florice | NA | NA | 11.62 | 13.26 |
| 12-424-35 | Isle_of_Jura | Florice | NA | NA | 12.24 | 13.88 |
| 12-424-36 | Isle_of_Jura | Florice | NA | NA | 13.48 | 15.13 |
| 12-424-41 | Isle_of_Jura | Florice | NA | NA | 10.43 | 12.06 |
| 12-424-42 | Isle_of_Jura | Florice | NA | NA | 9.1 | 10.73 |
| 12-424-43 | Isle_of_Jura | Florice | NA | NA | 8.78 | 10.38 |
| 12-425-07 | Isle_of_Jura | Jutlandia | 4 | 3.57 | 11.89 | 13.53 |
| 12-425-29 | Isle_of_Jura | Jutlandia | NA | NA | 12.04 | 13.68 |
| 12-425-36 | Isle_of_Jura | Jutlandia | NA | NA | 14.26 | 15.91 |
| 12-425-38 | Isle_of_Jura | Jutlandia | NA | NA | 14.97 | 16.63 |
| 12-425-42 | Isle_of_Jura | Jutlandia | NA | NA | 11.73 | 13.36 |
| 12-425-43 | Isle_of_Jura | Jutlandia | NA | NA | 12.02 | 13.66 |
| 12-426-02 | Isle_of_Jura | Rywal | NA | NA | 13.29 | 14.93 |
| 12-426-07 | Isle_of_Jura | Rywal | NA | NA | 13.04 | 14.68 |
| 12-426-14 | Isle_of_Jura | Rywal | NA | NA | 14.27 | 15.91 |
| 12-426-20 | Isle_of_Jura | Rywal | NA | NA | 11.65 | 13.28 |
| 12-426-23 | Isle_of_Jura | Rywal | NA | NA | 13.59 | 15.23 |
| 12-426-24 | Isle_of_Jura | Rywal | NA | NA | 12.85 | 14.48 |
| 12-426-28 | Isle_of_Jura | Rywal | NA | NA | 14.24 | 15.88 |
| 12-426-33 | Isle_of_Jura | Rywal | NA | NA | 16.35 | 18.01 |
| 12-426-41 | Isle_of_Jura | Rywal | NA | NA | 6.49 | 8.08 |
| 12-428-12 | 07-LJE-1 | Aventra | NA | NA | 13.91 | 15.56 |
| 12-428-21 | 07-LJE-1 | Aventra | NA | NA | 20.21 | 21.88 |
| 12-428-22 | 07-LJE-1 | Aventra | 6 | 5.57 | 17.61 | 19.28 |
| 12-428-27 | 07-LJE-1 | Aventra | 6 | 5.57 | 14.09 | 15.73 |
