# Supplementary material for: Genomic prediction of starch content and chipping quality in tetraploid potato using genotyping-by-sequencing
Source: Theor Appl Genet. 2017 Jul 13;130(10):2091–108. doi: 10.1007/s00122-017-2944-y (PMC5606954; doi:10.1007/s00122-017-2944-y)
Supplement: Supplementary file 2 — Supplementary material 2 (PDF 225 kb) [file 122_2017_2944_MOESM2_ESM.pdf]

Online Resource 2

Article title: Genomic prediction of starch content and chipping quality in tetraploid potato using genotyping-by-sequencing

Journal: Theoretical and Applied Genetics

Authors: Elsa Sverrisdóttir, Stephen Byrne, Ea Høegh Riis Sundmark, Heidi Øllegaard Johnsen, Hanne Grethe Kirk, Torben Asp, Luc Janss, and Kåre L. Nielsen

Corresponding author: Elsa Sverrisdóttir, Aalborg University, Department of Chemistry and Bioscience, Fredrik Bajers Vej 7H, 9220 Aalborg, Email: esv@bio.aau.dk; Telephone number: +45 5055 3092

List of cultivars in test panel

The following is a list of the cultivars and breeding clones selected for the test panel in the genomic selection project. The cultivars were grown, harvested and phenotyped over a number of years at the breeding station in Vandel, Denmark. The phenotypic data for chipping quality and starch content is listed as the mean over several years and replicates (raw average) and as the mean of corrected phenotypic data.

Cultivar	Chipping quality [scale 1-9]		Starch content [%]	
	Raw average	Corrected	Raw average	Corrected
00-DLS-1	5.5	4.99	22.24	23.4
01-EAZ-4	NA	NA	22.43	22.98
02-EMC-9	NA	NA	21.98	22.85
02-ENU-5	6	4.49	22.88	23.93
03-GAE-4	NA	NA	22.56	23.91
05-DLG-26	NA	NA	20.56	22.41
05-GUK-3	5.5	4.28	17.8	19.23
05-GUR-1	4.5	3.08	21.36	22.85
06-ECI-7	NA	NA	22.35	23.83
06-LDP-1	4.5	4.03	21.77	23.09
06-LEE-4	4	3.54	22.19	23.63
06-LEK-4	4.5	4.03	22.6	24
07-LPL-3	NA	NA	21.44	22.89
07-LPW-4	5.67	5.29	22.29	23.79
08-GJY-24	4.5	4.53	22.57	23.86
08-LWK-1	NA	NA	23.85	25.16
08-LWZ-1	NA	NA	23.06	24.39
08-LXB-1	NA	NA	20.82	22.1
08-LXB-10	NA	NA	21.8	23.1
08-LXC-3	NA	NA	21.6	22.96
08-LXC-4	NA	NA	21.01	22.37
08-LXD-2	NA	NA	21.65	23.03
08-LXE-2	NA	NA	21.73	23.02
08-LXJ-5	NA	NA	23.29	24.68
08-LZH-3	NA	NA	20.54	21.81
08-LZK-1	6	4.82	21.07	22.44
08-MAK-06	3	1.82	20.65	22.01
08-MAK-07	3	1.82	19.09	20.43
08-MAW-02	NA	NA	21.58	22.98
09-0-192-04	4.33	NA	17.61	19
09-GUT-23	2	0.82	23.47	24.95
09-GUT-25	NA	NA	23.19	24.68
09-LPV-6	NA	NA	22	23.48
09-MDI-7	NA	NA	21.26	22.71
09-MDP-12	NA	NA	22.04	23.51

09-MDP-6	NA	NA	21.75	23.21
09-MDP-9	NA	NA	23.12	24.6
09-MFD-6	5	4.62	21	22.45
93-CAG-1	4	4.43	21.13	22.06
93-CAL-3	3.5	3.93	19.51	20.71
96-BQD-56	NA	NA	22.01	22.78
Artana	NA	NA	20.41	21.48
Bonanza	6	5.81	19.66	21.45
Bruse	4	NA	18.12	19.14
Camel	2.25	NA	11.81	13.5
Canasta	5	3.98	20.98	22
Centaure	NA	NA	18.15	19.36
Danva	5	4.37	20.75	21.5
Festien	NA	NA	20.47	21.35
Jumbo	NA	NA	22.21	21.66
Karakter	NA	NA	18.69	20.13
Kardal	4.5	3.87	21.26	22.41
Magnum Bonum	NA	3.86	15.26	16.42
Odin	5	3.58	21.55	22.65
Oleva	4.4	3.95	18.89	19.97
Posmo	5	4.37	20.7	21.58
Ramses	NA	NA	20.11	20.63
Sa90-47-97	NA	NA	15.4	16.34
Seresta	4	2.49	21.87	22.65
Thor	4	3.53	23.16	24.55
Tidlig Rosen	NA	3.2	16.02	17.26
Wotan	4	3.53	22.65	24.03
Ydun	3.5	3.15	24.03	25.62
