# Supplementary material for: Genomic prediction of starch content and chipping quality in tetraploid potato using genotyping-by-sequencing
Source: Theor Appl Genet. 2017 Jul 13;130(10):2091–108. doi: 10.1007/s00122-017-2944-y (PMC5606954; doi:10.1007/s00122-017-2944-y)
Supplement: Supplementary file 3 — Supplementary material 3 (PDF 190 kb) [file 122_2017_2944_MOESM3_ESM.pdf]

Online Resource 3

Article title: Genomic prediction of starch content and chipping quality in tetraploid potato using genotyping-by-sequencing

Journal: Theoretical and Applied Genetics

Authors: Elsa Sverrisdóttir, Stephen Byrne, Ea Høegh Riis Sundmark, Heidi Øllegaard Johnsen, Hanne Grethe Kirk, Torben Asp, Luc Janss, and Kåre L. Nielsen

Corresponding author: Elsa Sverrisdóttir, Aalborg University, Department of Chemistry and Bioscience, Fredrik Bajers Vej 7H, 9220 Aalborg, Email: esv@bio.aau.dk; Telephone number: +45 5055 3092

Adapter and primer sequences

5’ Index adapter

1) 5’-ACACTCTTTCCCTACACGACGCTCTTCCGATCTTGCA
2) 5’-ACACTCTTTCCCTACACGACGCTCTTCCGATCTTGCGA
3) 5’-ACACTCTTTCCCTACACGACGCTCTTCCGATCTCGCTT
4) 5’-ACACTCTTTCCCTACACGACGCTCTTCCGATCTGGTTGT
5) 5’-ACACTCTTTCCCTACACGACGCTCTTCCGATCTCCAGCT
6) 5’-ACACTCTTTCCCTACACGACGCTCTTCCGATCTTATTTTT
7) 5’-ACACTCTTTCCCTACACGACGCTCTTCCGATCTCTTGCTT
8) 5’-ACACTCTTTCCCTACACGACGCTCTTCCGATCTACGACTAC

5’ Index adapter reverse complement

1) 5’-CWGTGCAAGATCGGAAGAGCGTCGTGTAGGGAAAGAGTGT
2) 5’-CWGTCGCAAGATCGGAAGAGCGTCGTGTAGGGAAAGAGTGT
3) 5’-CWGAAGCGAGATCGGAAGAGCGTCGTGTAGGGAAAGAGTGT
4) 5’-CWGACAACCAGATCGGAAGAGCGTCGTGTAGGGAAAGAGTGT
5) 5’-CWGAGCTGGAGATCGGAAGAGCGTCGTGTAGGGAAAGAGTGT
6) 5’-CWGAAAAATAAGATCGGAAGAGCGTCGTGTAGGGAAAGAGTGT
7) 5’-CWGAAGCAAGAGATCGGAAGAGCGTCGTGTAGGGAAAGAGTGT
8) 5’-CWGGTAGTCGTAGATCGGAAGAGCGTCGTGTAGGGAAAGAGTGT

3’ Index adapter

A) 5’-CWG AGATCGGAAGAGCACACGTCTGAACTCCAGTCACATCACGATCTCGTATGCCGTCTTCTGCTTG
B) 5’-CWG AGATCGGAAGAGCACACGTCTGAACTCCAGTCACCGATGTATCTCGTATGCCGTCTTCTGCTTG
C) 5’-CWG AGATCGGAAGAGCACACGTCTGAACTCCAGTCACTTAGGCATCTCGTATGCCGTCTTCTGCTTG
D) 5’-CWG AGATCGGAAGAGCACACGTCTGAACTCCAGTCACTGACCAATCTCGTATGCCGTCTTCTGCTTG
E) 5’-CWG AGATCGGAAGAGCACACGTCTGAACTCCAGTCACACAGTGATCTCGTATGCCGTCTTCTGCTTG
F) 5’-CWG AGATCGGAAGAGCACACGTCTGAACTCCAGTCACGCCAATATCTCGTATGCCGTCTTCTGCTTG
G) 5’-CWG AGATCGGAAGAGCACACGTCTGAACTCCAGTCACAGTCAAATCTCGTATGCCGTCTTCTGCTTG
H) 5’-CWG AGATCGGAAGAGCACACGTCTGAACTCCAGTCACACTTGAATCTCGTATGCCGTCTTCTGCTTG
I) 5’-CWG AGATCGGAAGAGCACACGTCTGAACTCCAGTCACGATCAGATCTCGTATGCCGTCTTCTGCTTG
J) 5’-CWG AGATCGGAAGAGCACACGTCTGAACTCCAGTCACTAGCTTATCTCGTATGCCGTCTTCTGCTTG
K) 5’-CWG AGATCGGAAGAGCACACGTCTGAACTCCAGTCACGGCTACATCTCGTATGCCGTCTTCTGCTTG
L) 5’-CWG AGATCGGAAGAGCACACGTCTGAACTCCAGTCACCTTGTAATCTCGTATGCCGTCTTCTGCTTG

3’ Index adapter reverse complement

A) 5’-CAAGCAGAAGACGGCATACGAGATCGTGATGTGACTGGAGTTCAGACGTGTGCTCTTCCGATCT
B) 5’-CAAGCAGAAGACGGCATACGAGATACATCGGTGACTGGAGTTCAGACGTGTGCTCTTCCGATCT
C) 5’-CAAGCAGAAGACGGCATACGAGATGCCTAAGTGACTGGAGTTCAGACGTGTGCTCTTCCGATCT
D) 5’-CAAGCAGAAGACGGCATACGAGATTGGTCAGTGACTGGAGTTCAGACGTGTGCTCTTCCGATCT
E) 5’-CAAGCAGAAGACGGCATACGAGATCACTGTGTGACTGGAGTTCAGACGTGTGCTCTTCCGATCT
F) 5’-CAAGCAGAAGACGGCATACGAGATATTGGCGTGACTGGAGTTCAGACGTGTGCTCTTCCGATCT

G) 5’-CAAGCAGAAGACGGCATACGAGATTTGACTGTGACTGGAGTTCAGACGTGTGCTCTTCCGATCT
H) 5’-CAAGCAGAAGACGGCATACGAGATTCAAGTGTGACTGGAGTTCAGACGTGTGCTCTTCCGATCT
I) 5’-CAAGCAGAAGACGGCATACGAGATCTGATCGTGACTGGAGTTCAGACGTGTGCTCTTCCGATCT
J) 5’-CAAGCAGAAGACGGCATACGAGATAAGCTAGTGACTGGAGTTCAGACGTGTGCTCTTCCGATCT
K) 5’-CAAGCAGAAGACGGCATACGAGATGTAGCCGTGACTGGAGTTCAGACGTGTGCTCTTCCGATCT
L) 5’-CAAGCAGAAGACGGCATACGAGATTACAAGGTGACTGGAGTTCAGACGTGTGCTCTTCCGATCT

PCR primer 1
5’-AATGATACGGCGACCACCGAGATCTACACTCTTTCCCTACACGACGCTCTTCCGATCT

PCR primer 2
5’-CAAGCAGAAGACGGCATACGAGAT
