# Supplementary material for: Genomic prediction of starch content and chipping quality in tetraploid potato using genotyping-by-sequencing
Source: Theor Appl Genet. 2017 Jul 13;130(10):2091–108. doi: 10.1007/s00122-017-2944-y (PMC5606954; doi:10.1007/s00122-017-2944-y)
Supplement: Supplementary file 5 — Supplementary material 5 (PDF 1386 kb) [file 122_2017_2944_MOESM5_ESM.pdf]

Online Resource 5

Article title: Genomic prediction of starch content and chipping quality in tetraploid potato using genotyping-by-sequencing

Journal: Theoretical and Applied Genetics

Authors: Elsa Sverrisdóttir, Stephen Byrne, Ea Høegh Riis Sundmark, Heidi Øllegaard Johnsen, Hanne Grethe Kirk, Torben Asp, Luc Janss, and Kåre L. Nielsen

Corresponding author: Elsa Sverrisdóttir, Aalborg University, Department of Chemistry and Bioscience, Fredrik Bajers Vej 7H, 9220 Aalborg, Email: esv@bio.aau.dk; Telephone number: +45 5055 3092

Supplementary figures

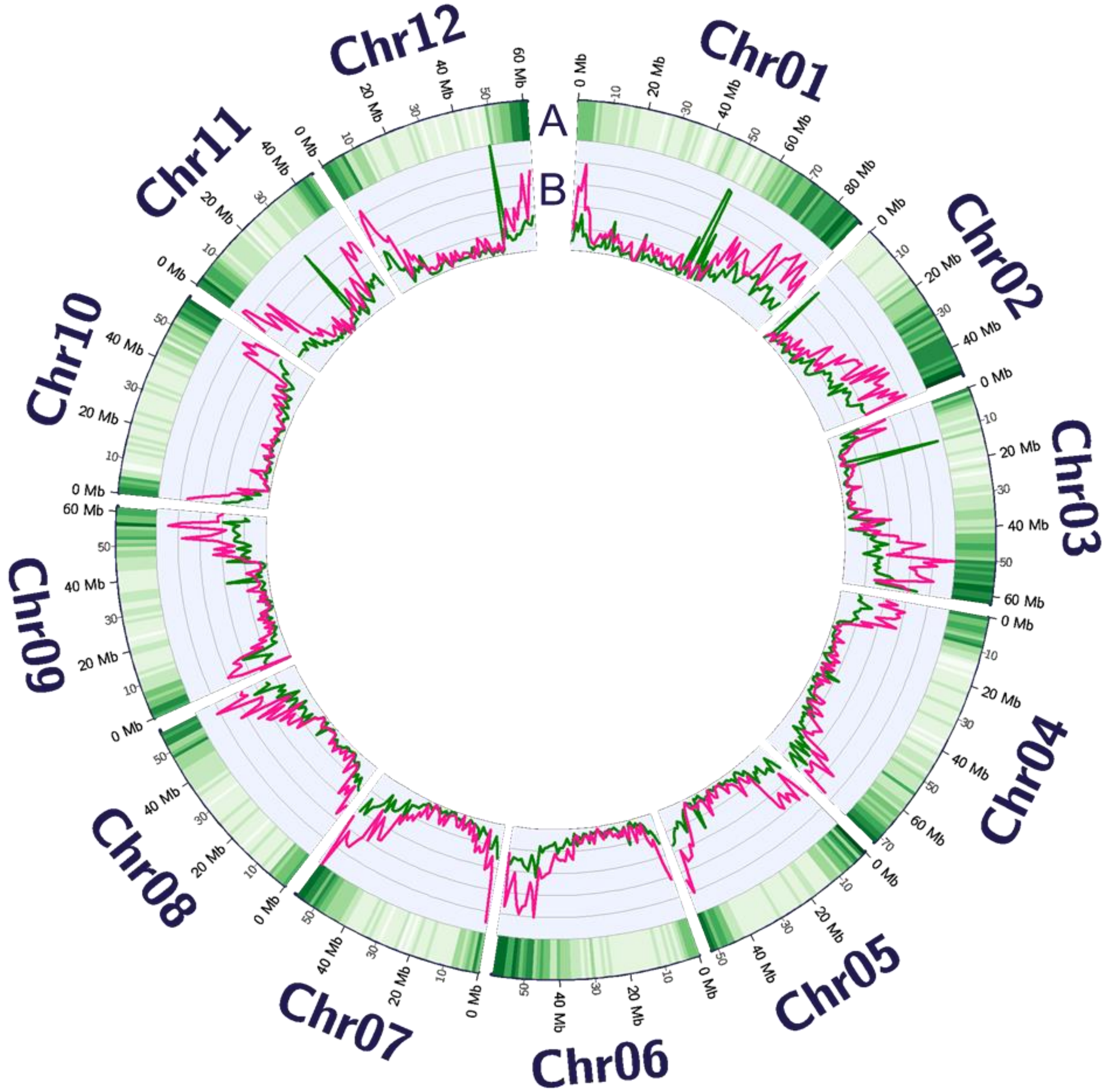

Fig. S1 A: Heat map of gene density ranging between 0 and 150 genes/Mb. B: Average coverage (green) and distribution of filtered markers (pink) in 1 Mb bins, normalised to the highest genome wide value.

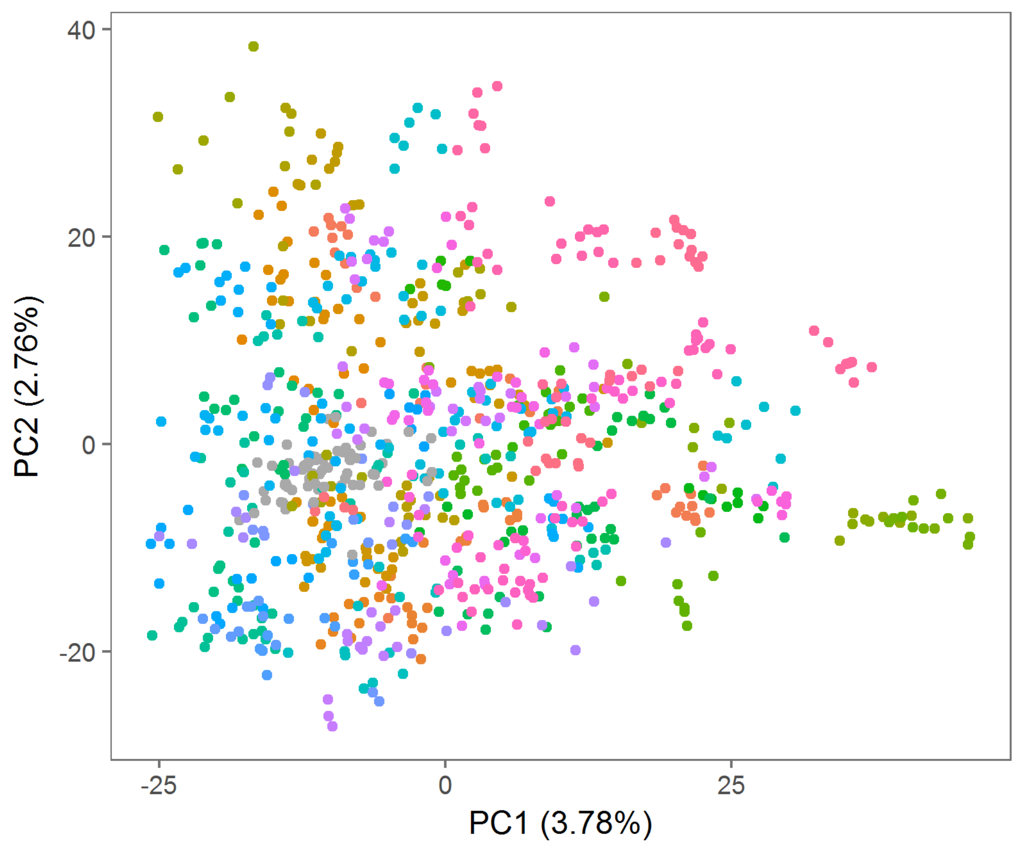

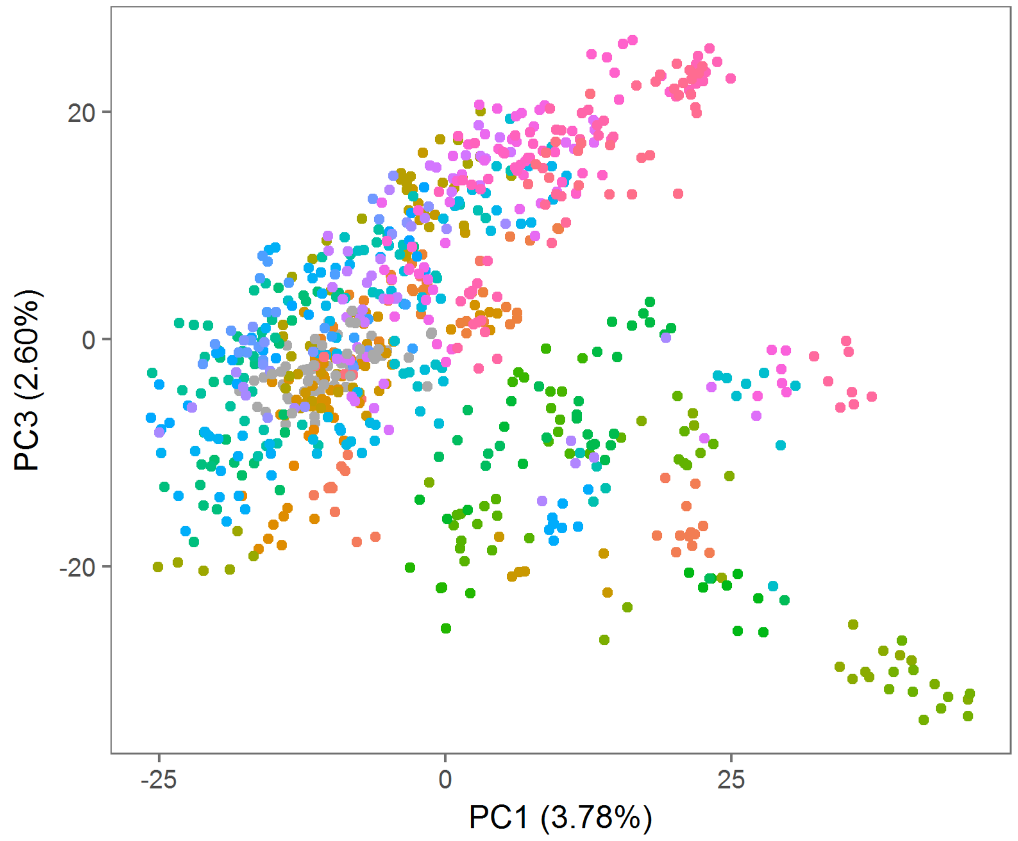

Fig. S2 Principal component analysis (PCA) of allele frequency data. Top: Principal component 1 and 2. Bottom: Principal component 1 and 3. Grey indicates individuals in the test panel, while the rest of the colours correspond to full-sibs in the MASPOT population.

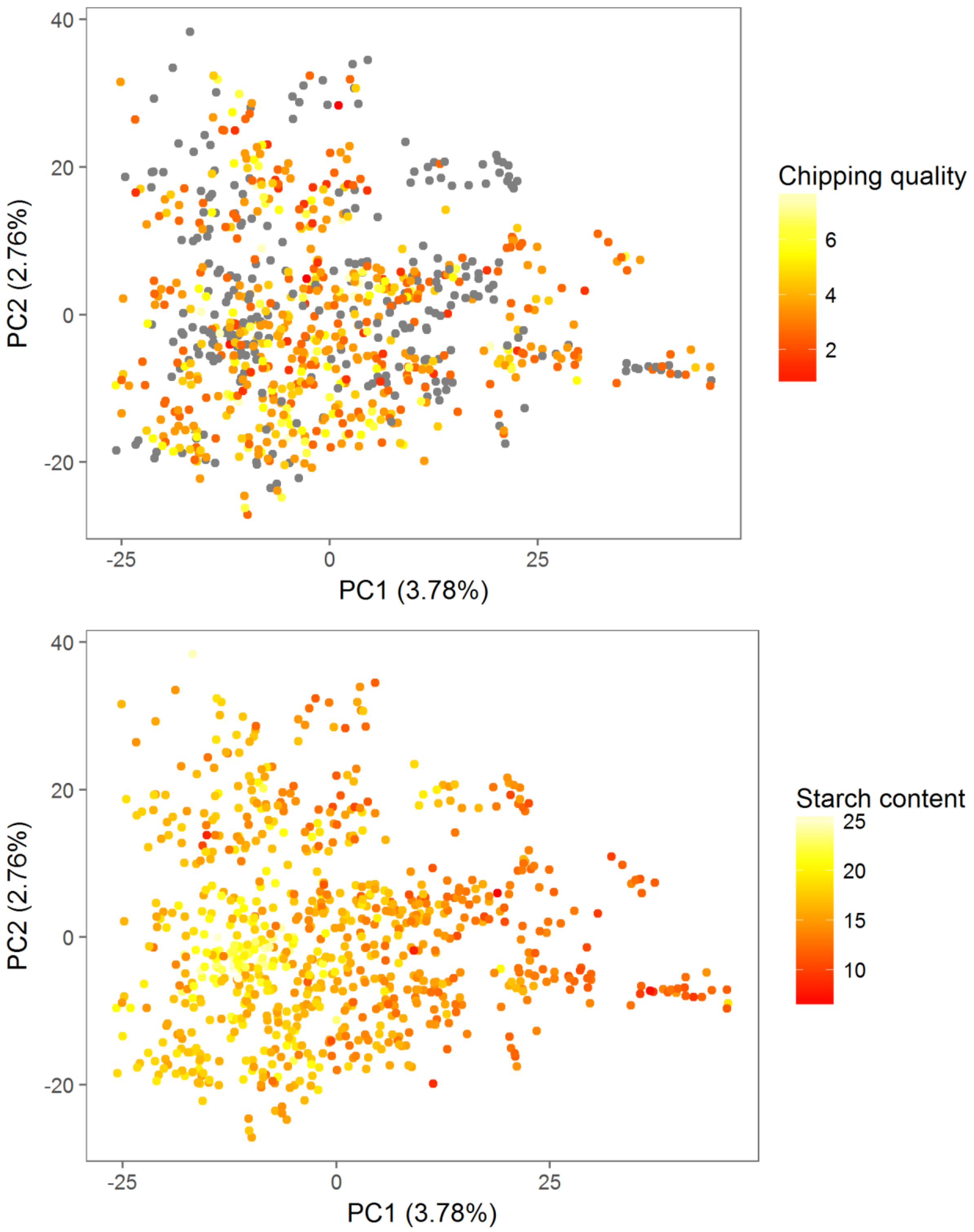

Fig. S3 Principal component analysis (PCA) of allele frequency data showing data points coloured according to chipping quality (top) or starch content (bottom). Grey colour refers to missing phenotype data. Principal component 1 and 2 are depicted.

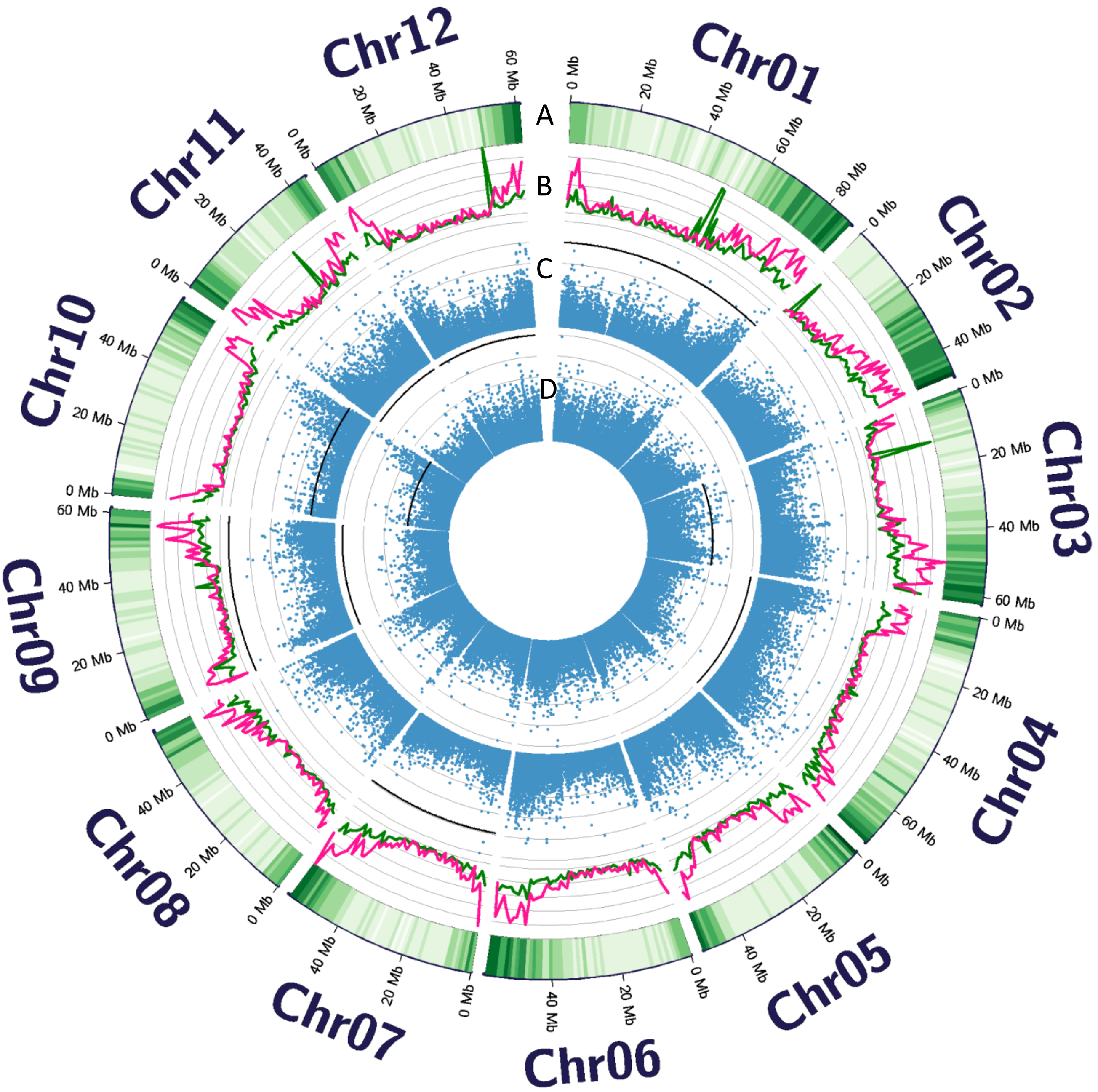

Fig. S4 GWAS analysis of GBS data for the MASPOT population. A) Heat map of gene density ranging between 0 and 150 genes/Mb. B) Average coverage (green) and distribution of filtered markers (pink) in 1 Mb bins, normalised to the highest genome wide value. C) GWAS results for starch content as -log10 transformed p-values normalised between 0 and 1. Black bars indicate FDR significance threshold for each chromosome. D) GWAS results for chipping quality as -log10 transformed p-values normalised between 0 and 1. Black bars indicate FDR significance threshold for each chromosome.

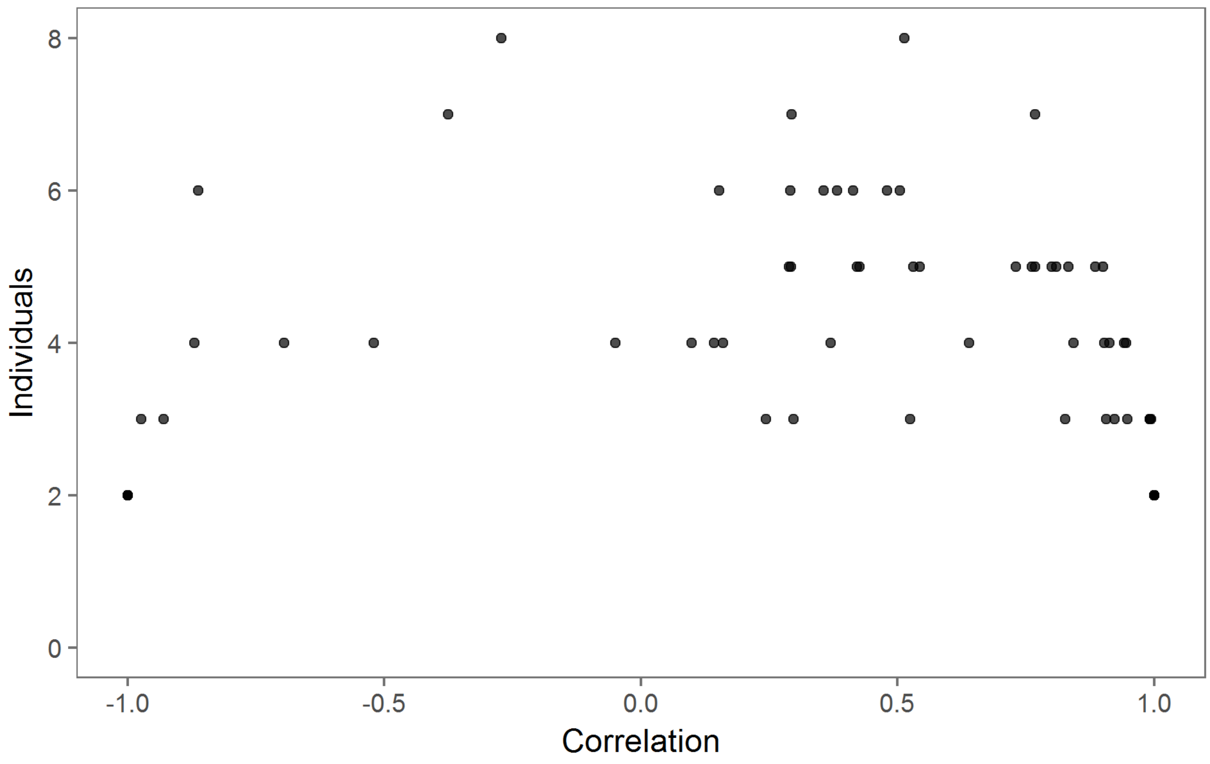

Fig. S5 Prediction correlation for chipping quality predictions plotted against number of test panel individuals in validation set. Prediction correlations were obtained for the test panel using the combined model.
